# How Supportive Care Shapes Women’s Childbirth Satisfaction: A Systematic Review

**DOI:** 10.3390/healthcare14142041

**Published:** 2026-07-08

**Authors:** Fortune Afi Agbi, Lulin Zhou, Eric Owusu Asamoah

**Affiliations:** 1 School of Management, Jiangsu University, Zhenjiang 212013, China; agbiafifortune@yahoo.com; 2 Faculty of Economics, University of Algarve, 8005-139 Faro, Portugal; andybruce1989@gmail.com

**Keywords:** supportive care, psychosocial childbirth experiences, women’s satisfaction, women-centred care, maternity care

## Abstract

**Background:** Despite strong clinical safety standards, many women continue to report negative or unsatisfactory childbirth experiences. Supportive care has gained attention as an important component of quality maternity services, yet evidence on its relationship with women’s satisfaction remains limited. **Objective:** This systematic review examined recent research on supportive care during childbirth and its association with women’s satisfaction and childbirth experiences. **Methods:** A systematic search of PubMed/MEDLINE, Web of Science, CINAHL, PsycINFO, and Scopus was conducted for peer-reviewed studies published between 2020 and 2025. Quantitative, qualitative, and mixed-methods studies examining supportive care during childbirth and maternal satisfaction were included. Twenty-five studies met the inclusion criteria. Data were synthesised narratively and supported by bibliometric co-occurrence mapping using VOSviewer to identify dominant thematic clusters. Included studies comprised randomised controlled trials, cross-sectional studies, quasi-experimental studies, qualitative interviews, and mixed-methods designs. **Results:** Supportive care was consistently associated with higher maternal satisfaction and more positive childbirth experiences. Common forms of support included emotional support, respectful communication, companionship, informational support, and autonomy-supportive care. Psychosocial factors such as emotional safety, perceived control, and mental wellbeing were reported as being associated with women’s experiences. **Conclusions:** Supportive care is an important component in the literature on women’s childbirth satisfaction. Emotional and relational aspects of care were frequently highlighted across studies. Integrating supportive practices into routine maternity care may contribute to more woman-centred approaches while maintaining clinical safety.

## 1. Introduction

Childbirth remains a universal human experience, with approximately 140 million births occurring globally each year [1]. In recent decades, remarkable progress has been made in reducing maternal and neonatal mortality through medical advancements, skilled birth attendance, and evidence-based clinical protocols [2,3]. Globally, maternal and neonatal mortality have declined over the past two decades, with the most substantial improvements occurring in low- and middle-income countries due to increased skilled birth attendance, emergency obstetric care, and evidence-based clinical protocols [1,4]. However, these gains are not universal.

Rising maternal mortality in several high-income countries, including the United States [5], highlights ongoing inequities and the need for context-specific maternity care improvements. This variation emphasises the importance of examining not only clinical outcomes but also the quality and experience of care across diverse health system contexts. However, this narrative of increasing clinical safety coexists with a notable paradox. Thus, despite safer births, a large proportion of women worldwide report dissatisfying, disempowering, or, in some cases, traumatic childbirth experiences [6,7]. Surveys across different healthcare systems show that between 20% and 40% of women describe their birth in negative terms, citing feelings of loss of control, fear, and a sense of being merely a “body” within a medicalized system [8,9,10]. This gap between technical success and negative experiential outcomes underscores that survival alone is an insufficient metric for quality maternity care.

In response, the global health paradigm has undergone a critical shift. The World Health Organization’s standard recommendations for intrapartum care for a positive childbirth experience explicitly reframe quality [11,12]. They define a positive experience as one that fulfills or exceeds a woman’s personal and sociocultural beliefs and expectations, emphasizing clinical and psychological outcomes. This framework establishes that the goal of maternity care is not merely a physically healthy mother and baby but also a woman who is respected, informed, empowered, and supported throughout the process. This shift from a survival-centric to an experience-centric model marks an important evolution in how quality is conceptualized and measured.

Historically, the assessment of maternity care has been dominated by clinical endpoints: mortality rates, incidence of hemorrhage or infection, Apgar scores, and rates of surgical intervention [6,13]. Even though these metrics are undeniably important, they capture only one dimension of care. They reflect a model of clinical competence, thus the technical skill and knowledge required to manage physiological processes and prevent harm. This focus, though lifesaving, has often inadvertently marginalized the human dimensions of birth. This can lead to care environments that excel clinically but may not fully address the psychosocial and emotional needs of laboring women [14,15].

Concurrently, there is growing recognition that experiential outcomes are valid, measurable, and important indicators of quality maternity care. Maternal satisfaction with childbirth is not merely a subjective or secondary outcome; rather, it is increasingly recognized as a key indicator of care quality with meaningful implications for both women and health systems [16,17]. Evidence demonstrates that positive childbirth experiences are associated with improved postpartum mental health, stronger mother–infant bonding, greater confidence in future reproductive decision-making, and more positive long-term perceptions of childbirth [18,19]. Conversely, dissatisfaction and childbirth-related traumatic stress may adversely affect family relationships, future healthcare-seeking behaviour, and trust in health systems [10,20]. These findings underscore the need for contemporary maternity care to move beyond ensuring clinical safety alone and to equally prioritize women’s experiences of care. Achieving this requires integrating high-quality clinical care with interpersonal practices that promote dignity, emotional support, and psychological well-being throughout childbirth. Within this context, supportive care may represent an important mechanism for bridging the gap between favourable clinical outcomes and positive childbirth experiences. Accordingly, this review examines the role of supportive care in shaping women’s satisfaction with childbirth.

Several important reviews have synthesized evidence on broader constructs such as respectful maternity care [21] or interpersonal quality [22,23]. These have been instrumental in emphasizing systemic issues of disrespect and abuse, and in establishing the importance of communication and respectful treatment [6,24]. Similarly, the Cochrane review on continuous support for women during childbirth provides robust evidence of its clinical benefits [25,26]. However, a focused synthesis that separates and examines the specific contribution of supportive care behaviours, separate from, yet interconnected with communication and respect, to the outcome of maternal satisfaction is lacking. This is a significant gap, as supportive care represents a highly implementable and trainable component of positive interpersonal interaction.

In response to these gaps and contextual shifts, this systematic review aims to synthesize the contemporary empirical evidence on the relationship between supportive care during childbirth and women’s satisfaction. We seek to move beyond establishing that support matters, to understanding the specifics of how, why, and under what conditions it influences the birth experience. This review seeks to clarify how supportive care contributes to women’s childbirth satisfaction beyond clinical competence. In doing so, it aims to inform maternity care practice, addressing three core questions:

1. How does supportive care during childbirth influence maternal satisfaction?
2. Which forms of supportive care are most strongly associated with satisfaction?
3. What pathways or mechanisms link supportive care to positive childbirth experiences?

## 2. Methods

This systematic review was conducted in accordance with the Preferred Reporting Items for Systematic Reviews and Preferred Reporting Items for Systematic Reviews and Meta-Analyses (PRISMA) 2020 guidelines [27]. The completed PRISMA 2020 checklist is provided as Supplementary File S1. The review adopted a structured and transparent approach to identify, appraise, and synthesize empirical evidence examining the relationship between supportive maternity care and maternal satisfaction.

This review was registered in PROSPERO (CRD420261385103), and the registration record, available at http://www.crd.york.ac.uk/PROSPERO/view/CRD420261385103 (accessed on 2 May 2026) outlines the key methodological decisions that guided the review. This work was supported by the National Natural Science Foundation of China (grant No. 71974079) awarded to (ZL). The funding body had no role in the study design, data collection, analysis, interpretation of findings, manuscript preparation, or decision to publish. Eligibility criteria were defined using the Population, Exposure, Outcome, and Study design (PEOS) framework [28] to ensure consistency and relevance.

Studies in this review were eligible if they involved women who received facility-based intrapartum or postpartum care. This included women who experienced vaginal or cesarean births in hospitals, maternity units, or other formal healthcare facilities. Studies focusing exclusively on antenatal care, postnatal care without an intrapartum component, or non-facility-based births were excluded. Studies involving birth companions, doulas, or healthcare providers were also eligible when they provided data directly relevant to supportive care during childbirth or contributed to understanding women’s childbirth experiences and satisfaction with care.

The exposure of interest was supportive care during childbirth. In this review, supportive care refers to the non-clinical and relational dimensions of intrapartum care that seek to address women’s emotional, informational, psychosocial, and interpersonal needs during labour and birth. It encompasses emotional reassurance, effective communication, continuous presence, advocacy, respect for dignity and autonomy, culturally sensitive care, and support provided by healthcare professionals, birth companions, or doulas. These dimensions are analytically distinct but often overlap in practice and collectively contribute to women’s experiences and evaluations of childbirth care. Eligible studies reported outcomes related to maternal satisfaction or women’s experiences of childbirth care. This included measures of overall satisfaction, satisfaction with care processes, perceived quality of support, and experiential outcomes reflecting women’s evaluations of their childbirth care. Studies involving birth companions or healthcare providers were included only when their findings directly informed these outcomes. Studies that reported only clinical or biomedical outcomes without reference to women’s satisfaction or experiences were excluded.

Quantitative, qualitative, and mixed-methods studies reporting primary empirical data were included. Quantitative studies included randomised controlled trials, quasi-experimental designs, cross-sectional studies, and other observational designs assessing associations between supportive care and maternal satisfaction. Qualitative studies explored women’s experiences and perceptions of supportive care during childbirth. Mixed-methods studies incorporating both quantitative and qualitative components were also eligible. Reviews, editorials, commentaries, protocols, conference abstracts, and opinion papers were excluded. Studies published in peer-reviewed journals between January 2020 and December 2025 to reflect contemporary empirical and conceptual developments in maternal care quality and satisfaction were eligible. Only studies published in English were included due to resource and feasibility constraints related to translation. Given the substantial methodological and clinical heterogeneity of the included studies, findings were synthesised narratively rather than quantitatively. Table 1 shows the summary of the inclusion and exclusion criteria in selecting relevant studies for this review.

### 2.1. Search Strategy

A wide-ranging literature search was conducted across multiple electronic databases to ensure broad coverage of relevant disciplines. The databases searched included PubMed/MEDLINE, Web of Science, CINAHL via EBSCO, PsycINFO and Scopus (Table 2), to ensure wide coverage of literature from medicine, midwifery, nursing, public health, psychology, and social sciences. These databases were selected because they index research relevant to intrapartum care, supportive care practices, and women’s childbirth experiences, consistent with the scope of this review. The search strategy combined controlled vocabulary terms (e.g., Medical Subject Headings [MeSH]) and free-text keywords related to maternity care, supportive care, and maternal satisfaction. Search terms were grouped into three main concepts:

(1) Maternity and childbirth care, (2) supportive care, and (3) maternal satisfaction or childbirth experience.

Boolean operators (“AND,” “OR”) and truncation were used to maximize sensitivity while maintaining relevance as shown in Table 2. Reference lists of included studies were screened manually (backward citation searching), and forward citation tracking was performed to identify additional relevant studies.

An example of the search strategy used in PubMed was as follows:

(“birth” OR “labour” OR “labor” OR “delivery” OR “childbirth” OR “maternity care”)

AND (“support *” OR “supportive care” OR “labour support” OR “continuous support” OR “birth support” OR “birth companion *” OR “birth companionship” OR “companion *” OR “companion support” OR “doula *” OR “emotional support” OR “respectful maternity care” OR “person-centred maternity care” OR “person centered maternity care”)

AND (“experience *” OR “birth experience” OR “childbirth experience” OR “satisfaction” OR “maternal satisfaction” OR “women’s experience *” OR “patient experience”).

The search strategy was adapted for each database according to its indexing and search functionalities (Table 2). Database-specific filters were applied where available, including limits on publication year, human subjects, and peer-reviewed articles. The timeframe (2020–2025) was selected to ensure conceptual alignment with contemporary definitions and recent evidence on supportive maternity care. Earlier research by Bohren, Vogel [29] and Hodnett, Gates [30] found that different studies defined and measured childbirth satisfaction and respectful care in very different ways. Because of this inconsistency, it was hard to compare results across studies. This methodological heterogeneity persisted until the publication of the World Health Organization (WHO) Intrapartum Care for a Positive Childbirth Experience guidelines in 2018, which introduced globally standardised principles emphasising respectful care, emotional support, effective communication, and companionship [12]. Subsequent contributions, including Bohren, Mehrtash [31] and Vedam, Stoll [32], further advanced validated domains and measurement tools for person-centred and respectful maternity care. As a result, studies published before 2020 often reflect earlier conceptual inconsistencies, whereas research from 2020 onward aligns more closely with current global standards.

In addition to these conceptual developments, the broader context of maternity care has also changed significantly in recent years. From 2020 onward, the COVID-19 pandemic introduced widespread restrictions on birth companions and healthcare personnel, removing key sources of support and creating a natural experiment in its absence [33]. During the same period, global attention to equity, trauma-informed care, and patient-reported experience measures (PREMs) intensified, further reshaping priorities in maternity care research and practice. A review covering 2020–2025 is therefore well positioned to synthesise evidence from this pivotal period and to inform practice and policy in a post-pandemic landscape where the importance of human connection in healthcare has become increasingly recognised. The final database search was conducted on 10 May 2026. In accordance with the prospectively registered PROSPERO protocol, studies published between 1 January 2020 and 31 December 2025 were eligible for inclusion. That is, all studies published in 2025 that met the eligibility criteria and were indexed in the searched databases at the time of the final search were considered.

All identified records were exported to EndNote software X8 (Clarivate Analytics, Philadelphia, PA, USA), and duplicates were removed prior to screening.

### 2.2. Study Selection

**Figure 1:**
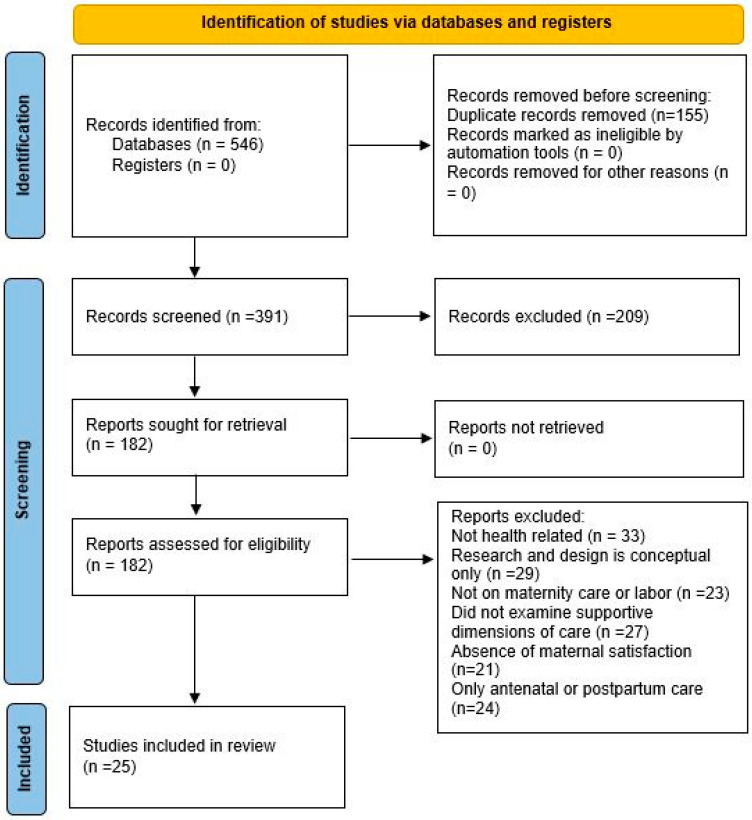
PRISMA flowchart.

Study selection followed a two-stage screening process. First, titles and abstracts were independently screened by two reviewers to assess potential eligibility based on the predefined criteria. Second, full texts of relevant articles were retrieved and independently reviewed for inclusion. Discrepancies at either stage were resolved through discussion and consensus, with consultation of the third reviewer. During screening, 209 studies were excluded because they did not meet the eligibility criteria. Other studies were removed for reasons such as being unrelated to health, lacking empirical data, not focusing on maternity care or labour. Additional studies were removed because they were not examining supportive dimensions of care, or not including maternal satisfaction outcomes. Studies that focused solely on antenatal or postpartum care were also excluded. This rigorous process ensured that only relevant studies were included in this review. Twenty-five (25) studies met the inclusion criteria. The study selection process is documented using a PRISMA flow diagram (Figure 1).

Data were extracted using a standardized extraction form developed for this review. Extracted information included: author(s), year of publication, country or region, study design, sample characteristics, definitions and measures of supportive care, maternal satisfaction outcomes and key findings. Data extraction was conducted independently by two reviewers to ensure accuracy and completeness. The methodological quality of the 25 included studies was assessed using the Mixed Methods Appraisal Tool (MMAT) [34] based on study design (Table 3). The MMAT was selected because it allows appraisal of qualitative, quantitative, and mixed-methods studies within a single framework. Given the heterogeneity of included qualitative, quantitative, and mixed-methods studies, MMAT was selected to ensure consistent appraisal across study designs. MMAT was used to assess methodological quality at the study level. This does not represent a formal assessment of certainty of evidence, and no GRADE or CERQual framework was applied.

Quality appraisal was conducted by one reviewer and checked independently by a second reviewer, with any discrepancies resolved through discussion to ensure consistency and accuracy. Overall, the included studies demonstrated generally good methodological quality across qualitative, quantitative, and mixed-methods designs. Most studies met the majority of MMAT criteria, indicating acceptable methodological rigor. Common methodological strengths included appropriate study designs, adequate sampling strategies, and use of validated measurement tools. Some studies exhibited limitations, particularly related to randomisation procedures, blinding, and control of confounding in quantitative designs, as well as limited reflexivity reporting in a small number of qualitative studies.

No studies were excluded based on methodological quality, as all met the minimum eligibility criteria for inclusion. The results of the MMAT appraisal were used to inform interpretation of findings rather than to generate exclusion or strict categorical quality rankings. During narrative synthesis, findings were interpreted in light of study design, methodological quality, and consistency across studies. Greater emphasis was placed on recurring findings observed across multiple studies and study designs rather than on individual studies alone. Where studies had methodological limitations, these were considered during synthesis and discussion of the evidence base.

Because several studies addressed multiple dimensions of supportive care, findings were sometimes synthesised across overlapping themes depending on their relevance to specific research questions. This approach ensured that all relevant evidence was incorporated while maintaining alignment with the review objectives.

### 2.3. Definition of Clusters

To examine conceptual relationships within the evidence base and obtain a macro-level view of how key ideas are organised across the included studies, all papers that met the final inclusion criteria were exported from EndNote X8 (Clarivate Analytics, Philadelphia, PA, USA) in RIS format and imported into VOSviewer software version 1.6.20 (Centre for Science and Technology Studies, Leiden University, Leiden, the Netherlands). Terms were extracted from titles and abstracts using the full counting method, with a minimum occurrence threshold of five to ensure that only substantively represented concepts were included. No thesaurus file was used. Terms were analysed as exported from the bibliographic database, with duplicate records removed and obvious non-informative terms excluded during data preparation. Clusters were defined based on shared conceptual meanings, allowing related terms to be grouped into coherent thematic categories. The VOSviewer algorithm organised these terms into five primary clusters, each represented by a distinct colour. These clusters were used to identify dominant themes and highlight conceptual gaps within the literature on supportive care and women’s childbirth satisfaction within the included studies. The VOSviewer analysis was not used to generate or replace themes identified through the narrative synthesis. Rather, it served as a complementary descriptive tool to visualize conceptual relationships among key terms and to provide a field-level perspective on thematic patterns identified in the included studies.

### 2.4. Research Term Cluster

**Figure 2:**
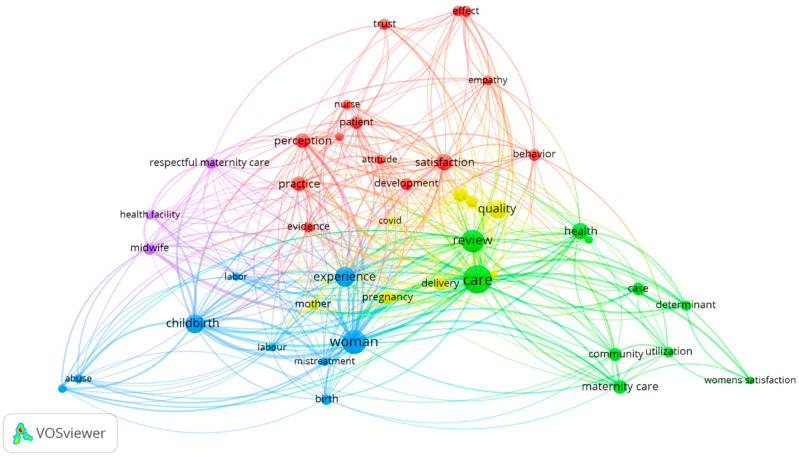
VOSviewer term co-occurrence network showing thematic clusters.

VOSviewer was applied to create a bibliometric co-occurrence map of research terms (Figure 2). The resulting map displayed five clusters, with closely related terms positioned nearer to each other, indicating stronger conceptual linkages. Although bibliometric co-occurrence mapping is often used with large datasets, it is equally appropriate for focused bodies of literature, as it can visually reveal how key concepts cluster within a defined evidence base. In this review, the co-occurrence analysis was used to map conceptual patterns within the included studies, providing a complementary, data-driven perspective alongside the narrative synthesis. The size of each node and label reflects the frequency and relative prominence of each term across the included papers.

The visualization shows that terms related to *maternity care, childbirth, pregnancy, women, labour, and satisfaction* are highly recurrent and centrally positioned, indicating a strong research emphasis on women’s experiences and outcomes of care. Concepts such as *satisfaction, perception, mistreatment, and social support* appear closely interconnected, underscoring the importance of psychosocial and relational dimensions in shaping childbirth experiences. Terms related to *emotional support, informational support, and partner or midwife support* further highlight the centrality of supportive care in the literature. Broader system-level concepts, including quality of care and health services, also appear, reflecting the influence of organisational and structural factors on women’s experiences.

In contrast, determinants of care and contextual influences such as COVID-19 appear less densely connected, suggesting that these systemic factors are less prominent within the included evidence base. The map provides an exploratory visual representation that women’s childbirth satisfaction is most frequently examined through experiential, interpersonal, and psychosocial lenses, with biomedical or clinical perspectives playing a more limited role.

The bibliometric map provides a field-level perspective that complements the narrative synthesis. While the narrative synthesis identifies themes within the included studies, the co-occurrence map demonstrates how these themes cluster conceptually, highlighting areas where research is less developed. Examining sparsely populated clusters reveals opportunities for future research to further integrate supportive and woman-centred care perspectives into maternity services. These insights can guide researchers, practitioners, and policymakers seeking to strengthen maternity care practices that prioritise respectful, supportive, and satisfying childbirth experiences. Because the co-occurrence analysis was based on a relatively small corpus of 25 studies, the findings should be interpreted as exploratory rather than definitive.

Although the included studies reported different but related outcomes, including maternal satisfaction, childbirth experience, emotional wellbeing, perceived control, and birth perception, these outcomes were synthesised narratively because they represent interrelated experiential dimensions of supportive intrapartum care. Outcomes were grouped according to their conceptual relevance to the review questions rather than according to identical measurement instruments. This approach enabled findings from diverse study designs to be integrated while maintaining consistency with the review objectives.

## 3. Results

### 3.1. Demographic Characteristics

Across the 25 included studies, demographic characteristics were broadly consistent. The studies represented 16 countries across high, middle, and low-income countries (LMICs), offering a geographically diverse evidence base that allows examination of supportive care across varied maternity care environments. The highest representation came from Turkey, Sweden, and the Netherlands, each contributing three (3) studies, followed by Oman, China, and Kenya, which contributed two (2) studies each. Single studies were conducted in Uganda, Ethiopia, South Africa, Pakistan, Korea, Israel, India, Nigeria, Rwanda, and Iran, reflecting a wide global distribution of evidence. All studies were conducted within a single country; however, several were multicentric (*n* = 8), drawing participants from multiple hospitals, maternity units, or maternity care organisations within the same country. The remaining studies were single-site, typically based in one hospital, clinic, or maternity unit. Table 4 shows the summary of included study characteristics.

### 3.2. Participants

Across the twenty-five (25) studies included in this review, participant groups varied according to study aims and methodological approaches. Most studies (*n* = 22) recruited women in labour or postpartum, while five (5) studies included birth companions, and three (3) involved maternity care providers as support. Maternal age was reported in 20 studies and ranged from 18 to 45 years, with mean ages typically between 24 and 32 years, indicating a predominantly young adult population across settings. Parity was clearly reported in 23 studies. Four RCTs and one quasi-experimental study focused exclusively on primiparous women, while the remaining studies included mixed-parity samples. Gestational age was reported in 20 studies, with 17 restricting inclusion to term pregnancies (≥37 weeks). Mode of birth was documented in 19 studies, with vaginal birth rates ranging from 60% to 85%, depending on the population and facility type.

Educational attainment was reported in 21 studies and varied by region, with higher levels in high-income settings such as Sweden, the Netherlands, and Israel, and more mixed or lower levels in LMIC settings such as Nigeria, Pakistan, Ethiopia, and India. Marital status was reported in 17 studies, and in 14 of these, 70–95% of participants were married or cohabiting. Sample sizes ranged from small qualitative cohorts (*n* = 10–60) to large cross-sectional surveys exceeding 4000 participants, with the largest study including 12,239 women. A small number of studies also described migrant women, women giving birth during COVID-19, or other socially vulnerable groups, highlighting how structural and social factors intersect with access to support during labour and birth. Where participant descriptions were limited or ambiguously reported, classifications were inferred based on each study’s stated objectives, methods, and data collection procedures to ensure consistent categorisation across the review.

### 3.3. Mapping of Studies to the Research Questions

The sample of twenty-five (25) articles was analyzed, with results organized into three tables corresponding to the research questions (RQ). Each table includes the relevant studies, their contexts, and key characteristics. This method enhances visualization and connects the findings directly to the specific research questions.

- *RQ1: How does supportive care during childbirth influence maternal satisfaction?*

#### 3.3.1. Reasoning for Systematizing Table 5

These studies (Table 5) were synthesised under Research Question One because they describe how supportive care is associated with women’s reported childbirth experiences and satisfaction across diverse settings. Using quantitative, qualitative, and mixed-methods designs, these studies consistently show that emotional support, respectful care, and the presence of support persons are associated with greater comfort, trust, and more positive childbirth experiences. Conversely, the absence of supportive and respectful care is commonly linked to lower satisfaction. Across all 25 included studies, supportive care was generally associated with higher maternal satisfaction. In randomised trials, women who received continuous midwife or companion support described lower fear, anxiety, and physiological stress (e.g., cortisol) and reported more positive perceptions of childbirth [40,41,43]. Findings from quasi-experimental [47] and observational studies [46] aligned with this pattern, reporting associations between supportive care and higher satisfaction, improved comfort, and reduced need for interventions. For example, continuity of midwifery care was linked to higher satisfaction and fewer interventions [44], while single-room labour environments increased perceived control and satisfaction [50].

**Table 5:** Studies included in the systematic review, relevant to answer research question 1.

Author and Year	Country	Design	Sample Size	Supportive Care Focus	Outcomes Related to Satisfaction/Experience
Stjernholm, Charvalho [40]	Sweden	RCT	59	Continuous midwife support	Continuous support led to shorter labour and lower cortisol, indicating reduced stress and more positive birth experience.
Balcik Colak, Akin [41]	Turkey	RCT	104	Companion support	Companion support increased comfort and satisfaction with childbirth.
Liu, Li [48]	China	Quantitative cross-sectional	4192	Midwife-led care	Reported high satisfaction with midwife-led care; predictors of satisfaction were identified.
Scheenen, Timmers [35]	Netherlands	Qualitative	14	Continuous care by maternity care assistants	Women described positive experiences of continuous care, while also identifying barriers to fully person-centred support.
Wanyenze, Byamugisha [36]	Uganda	Qualitative	10	Birth companions	Companions provided emotional and physical support; women described this as helpful, though some harmful behaviours were also noted, shaping overall experience.
Alwahaibi, Al-Julandani [49]	Oman	Quantitative cross-sectional	214	Birth companions	96.7% of women found companions helpful; encouraging words were the most common support, contributing to positive birth experiences.
Schytt, Wahlberg [42]	Sweden	RCT	164	Community-based doula (CBD) support for migrant women	CBD support did not improve satisfaction or wellbeing compared with standard care; an important null finding.
Purandare, Ådahl [58]	Sweden	Mixed methods	82 + 12	Community-based doulas	Doulas met women’s emotional, informational, and physical needs, contributing to more positive and supported birth experiences.
Jeong, Kim [44]	Korea	Non-randomized	63	Continuity of midwifery care	Continuity of care was associated with lower interventions and higher satisfaction with childbirth.
Demirel, Kaya [50]	Turkey	Quantitative cross-sectional	686	Single vs. shared rooms	Single rooms were associated with greater perceived support and control and higher satisfaction compared with shared rooms.
Nakphong, Afulani [45]	Kenya	Quantitative non-randomized	1138 women + 606 support persons	Structured involvement of support persons (PC-ISP)	High integration of support persons was associated with higher PCMC scores, greater satisfaction, and better adherence to WHO-recommended practices.
Hagaman, Rodriguez [37]	Ethiopia	Qualitative	41	Supportive communication and family support	Women’s satisfaction was shaped by supportive communication and family support; lack of these contributed to negative experiences.
Van der Pijl, Verhoeven [51]	Netherlands	Quantitative cross-sectional	12,239	Experience of care (support, communication, autonomy)	Lack of support, communication, and autonomy was strongly associated with negative birth experiences. Shows that absence of respectful/supportive care significantly reduces satisfaction.
Alwahaibi, Alajaimi [52]	Oman	Quantitative cross-sectional	444 women + 444 Women with companions	Companions during labour	84.1% of women felt calmer with companions; mothers were the most preferred companions; overall high satisfaction with support.
Li, Xiao [53]	China	Quantitative cross-sectional	230	Partner support	Emotional and informational support gaps were associated with increased trauma; low partner involvement was linked to more negative birth experiences.
Muhayimana, Kearns [38]	Rwanda	Qualitative	30	Respectful maternity care	Compassionate care, autonomy, timely care, privacy, and a supportive environment were central to positive childbirth experiences.
Van der Pijl, Kasperink [54]	Netherlands	Quantitative cross-sectional	767	Respectful care domains	Respect, communication, confidentiality, and autonomy scored high but not optimal, indicating room for improvement in women’s experiences.
Dahan and Goldberg [55]	Israel	Quantitative survey	788	Private midwife/doula one-on-one support	One-on-one support was associated with more natural births and a higher “flow state”, reflecting a more positive and satisfying birth experience.
Dubey, Sharma [46]	India	Comparative observational	150	Birth companionship	Companionship increased spontaneous vaginal birth, reduced augmentation, and increased satisfaction with childbirth.
Ihudiebube-Splendor, Enwereji-Emeka [59]	Nigeria	Mixed methods	280 + 15	Social support during COVID-19	Social support from partners, midwives, and family increased satisfaction, while COVID-19 restrictions created barriers to support and worsened experiences.
Akçay and Alan [43]	Turkey	RCT	72	Empathic communication training	Empathic communication training increased positive birth perception and reduced fear, contributing to more satisfying experiences.
Summerton, Mtileni [39]	South Africa	Qualitative	73	Birth companions’ perspectives	71/73 companions reported positive experiences; they valued respectful care, though some witnessed mistreatment, which affected women’s experiences.
Hameed, Khan [56]	Pakistan	Quantitative cross-sectional	314	Companion support	High companion support was associated with reduced mistreatment and lower postpartum anxiety/depression; informational support was the strongest protective factor.
Shahbazi Sighaldeh, Azadpour [47]	Iran	Quasi- experimental	150	Doula and trained lay companion	Doula and trained lay companion support reduced labour duration; lay companion support reduced anxiety, and doula support increased satisfaction.
Kiti, Prata [57]	Kenya	Quantitative cross-sectional	865	Continuous support	Continuous support increased PCMC scores by +4 points, with a strong adjusted association, indicating better person-centred and more satisfying care.

Large cross-sectional studies further reinforced these relationships, identifying communication, respect, autonomy, and emotional reassurance as strong predictors of satisfaction, with deficits in these areas contributing to negative experiences [48,51,52,57]. Qualitative studies echoed these findings, with women describing how emotional reassurance, respectful communication, and the presence of a trusted companion shaped their sense of safety, dignity, and emotional well-being [35,36,37,38,39]. Even studies reporting null effects, such as community-based doula support not improving satisfaction among migrant women added nuance by highlighting that supportive care must be contextually appropriate to be effective [42].

One mixed-methods study conducted during the COVID-19 pandemic reported that women who received social support from partners, midwives, and family described higher satisfaction, while pandemic-related restrictions on support persons and reduced interpersonal contact were associated with lower satisfaction, highlighting the continued importance of supportive care even in crisis contexts [59].

Overall, the results show that supportive care, whether emotional, informational, physical, or relational plays an important role in shaping women’s satisfaction with childbirth. Together, these findings position supportive care as a key determinant of maternal satisfaction across diverse settings.

- *RQ2: Which forms of supportive care are most strongly associated with satisfaction?*

#### 3.3.2. Reasoning for Systematizing Table 6

These studies (Table 6) were synthesised under Research Question Two because they explicitly disaggregate supportive care into its core components and examine how each relates to maternal satisfaction. Across the 22 studies that identified specific types of supportive care, four forms appeared most consistently: emotional support, informational support, physical support, and advocacy or communication support. Emotional support was the most frequently described, with women emphasising reassurance, encouragement, and an empathic presence as important to their sense of calm and confidence during labour [35,36,39,40,41,43,46,47,49,52,56,58]. Informational support, including clear explanations, guidance, and involvement in decision-making was also strongly associated with satisfaction [44,45,53,56,58]. This was particular in studies using the PCMC framework, where communication and autonomy domains aligned closely with positive experiences [45,53,57]. For example Kiti, Prata [57] reported that continuous support increased PCMC by +4 points. Physical support, such as massage, positioning assistance, and breathing guidance, was associated with greater comfort and coping in randomised trials, qualitative and observational studies [36,39,40,41,46,55]. Advocacy and communication support were especially salient in settings where mistreatment or disrespect were concerns [37,51,54]; companions and doulas often acted as intermediaries, and women described feeling more protected and respected in their presence [37,51,54,58].

**Table 6:** Studies included in the systematic review, relevant to answer research question 2.

Study	Country	Design	Sample Size	Forms of Support Identified	Detailed Outcomes (Specific to RQ2)
Alwahaibi, Al-Julandani [49]	Oman	Quantitative cross-sectional	214	Emotional	Encouraging words were the most common support; 96.7% of women found companions helpful.
Alwahaibi, Alajaimi [52]	Oman	Quantitative cross-sectional	444 + 444	Emotional	84.1% of women felt calmer with companions; mothers were the most preferred support persons.
Purandare, Ådahl [58]	Sweden	Mixed methods	82 + 12	Emotional, informational, advocacy	Doulas met women’s emotional, informational, and physical needs, improving perceived support.
Wanyenze, Byamugisha [36]	Uganda	Qualitative	10	Emotional, physical	Companions provided emotional reassurance and physical comfort, reducing fear.
Dubey, Sharma [46]	India	Comparative Observational	150	Emotional, physical	Companionship increased spontaneous vaginal birth, reduced augmentation, and improved satisfaction.
Stjernholm, Charvalho [40]	Sweden	RCT	59	Emotional, physical	Continuous support reduced stress (lower cortisol) and improved coping.
Balcik Colak, Akin [41]	Turkey	RCT	104	Emotional, physical	Companion support increased comfort and satisfaction.
Akçay and Alan [43]	Turkey	RCT	72	Emotional (empathic communication)	Empathic communication training reduced fear and improved birth perception.
Muhayimana, Kearns [38]	Rwanda	Qualitative	30	Compassion, autonomy, privacy	Compassionate care and autonomy were central to positive experiences.
Li, Xiao [53]	China	Cross-sectional	230	Informational	Gaps in emotional and informational support increased trauma and reduced satisfaction.
Nakphong, Afulani [45]	Kenya	Non-randomized	1138 + 606	Informational, communication, autonomy	High integration of support persons increased PCMC, autonomy, and satisfaction.
Kiti, Prata [57]	Kenya	Cross-sectional	865	Communication, autonomy	Continuous support increased PCMC by +4 points (strong adjusted association).
Van der Pijl, Verhoeven [51]	Netherlands	Quantitative cross-sectional	12,239	Communication, autonomy	Lack of communication and autonomy strongly predicted negative experiences.
Van der Pijl, Kasperink [54]	Netherlands	Quantitative cross-sectional	767	Communication, confidentiality	Respect, communication, and confidentiality scored high but not optimal.
Hameed, Khan [56]	Pakistan	Cross-sectional	314	Informational, emotional	Informational support was the strongest predictor of reduced mistreatment and anxiety.
Jeong, Kim [44]	Korea	Non-randomized	63	Informational continuity	Continuity improved communication and reduced interventions.
Scheenen, Timmers [35]	Netherlands	Qualitative	14	Emotional security	Women valued emotional presence and continuity.
Summerton, Mtileni [39]	South Africa	Qualitative	73	Emotional, physical	Companions valued respectful care; some witnessed mistreatment.
Hagaman, Rodriguez [37]	Ethiopia	Qualitative	41	Respectful communication	Supportive communication shaped satisfaction; lack of it worsened experiences.
Dahan and Goldberg [55]	Israel	Survey	788	One-on-one continuous support	One-on-one support increased natural birth and “flow state.”
Shahbazi Sighaldeh, Azadpour [47]	Iran	Quasi-experimental	150	Emotional, physical	Doula support increased satisfaction; lay companion reduced anxiety.
Demirel, Kaya [50]	Turkey	Quantitative cross-sectional	686	Environmental (privacy, control)	Single rooms increased perceived support, control, and satisfaction.

Across diverse contexts, studies consistently reported that women who received these forms of support, either individually or in combination, described more positive and satisfying childbirth experiences. For example Alwahaibi, Alajaimi [52] reported that 84.1% of women felt calmer with companions and mothers were the most preferred support persons. Conversely, gaps in emotional or informational support were associated with fear, trauma, and dissatisfaction [50,53,57]. The findings highlight emotional reassurance, companion presence, empathic communication, respectful interaction, and autonomy as distinct yet interconnected dimensions of supportive care. By examining specific support modalities rather than treating supportive care as a single construct, these studies provide clearer insight into how different forms of support relate to maternal satisfaction.

- *RQ3: What pathways or mechanisms link supportive care to positive childbirth experiences?*

#### 3.3.3. Reasoning for Systematizing Table 7

These studies (Table 7) were synthesised under Research Question Three because they move beyond identifying forms of supportive care and explore how women describe the ways support relates to their childbirth experiences. Across the 20 studies that discussed underlying processes, several mechanisms were identified. The most frequently reported mechanism involved reductions in fear, anxiety, and stress. This was reflected in both physiological indicators and women’s accounts of feeling calmer and more secure when supported [36,40,41,43,44,46]. Another commonly described mechanism was enhanced autonomy and participation in decision-making [38,50]. Women reported a stronger sense of involvement when support persons were present and when communication was clear [38,45,57]. Improved communication and understanding also emerged as key mechanisms [37,51,54,57]. Thus, women described less uncertainty and greater trust when they received timely information and explanations, while communication gaps were frequently associated with negative or distressing experiences.

**Table 7:** Studies included in the systematic review, relevant to answer research question 3.

Study	Country	Design	Sample Size	Mechanisms Identified	Detailed Outcomes (Specific to RQ3)
Stjernholm, Charvalho [40]	Sweden	RCT	59	Stress reduction	Continuous support was associated with lower cortisol levels and reduced stress.
Akçay and Alan [43]	Turkey	RCT	72	Fear reduction	Empathic communication was associated with reduced fear and improved emotional readiness.
Balcik Colak, Akin [41]	Turkey	RCT	104	Coping, fear reduction	Companion reassurance was associated with reduced fear and improved coping.
Dubey, Sharma [46]	India	Observational	150	Anxiety reduction	Companionship was linked to reduced anxiety and improved labour progress.
Wanyenze, Byamugisha [36]	Uganda	Qualitative	10	Fear reduction	Emotional and physical support was associated with reduced fear and increased comfort.
Jeong, Kim [44]	Korea	Non-randomized	63	Continuity, reduced anxiety	Continuity of care was linked to lower interventions and reduced anxiety.
Purandare, Ådahl [58]	Sweden	Mixed	82 + 12	Emotional safety, trust	Doulas involvement was linked to increased emotional safety and trust in providers.
Alwahaibi, Alajaimi [52]	Oman	Cross-sectional	444 + 444	Emotional calming	The presence of companions was associated with women feeling calmer and more secure.
Nakphong, Afulani [45]	Kenya	Non-randomized	1138 + 606	Autonomy, decision-making	Structured involvement was associated with higher autonomy and improved PCMC.
Kiti, Prata [57]	Kenya	Cross-sectional	865	Autonomy, communication	Continuous support was linked to +4 (higher) PCMC points and improved autonomy.
Van der Pijl, Verhoeven [51]	Netherlands	Descriptive	12,239	Communication	Poor communication was strongly associated with negative childbirth experiences.
Van der Pijl, Kasperink [54]	Netherlands	Cross-sectional	767	Respect, communication	Respectful treatment and good communication were central to positive childbirth experiences.
Li, Xiao [53]	China	Cross-sectional	230	Information, trauma	Information gaps were associated with increased trauma and more negative childbirth experiences.
Hagaman, Rodriguez [37]	Ethiopia	Qualitative	41	Communication, respect	Supportive communication was linked to higher satisfaction, while mistreatment was associated with negative experiences.
Scheenen, Timmers [35]	Netherlands	Qualitative	14	Emotional security	Continuous care was linked to emotional security and trust.
Summerton, Mtileni [39]	South Africa	Qualitative	73	Emotional safety	Companions were described as providing emotional safety; some women also reported witnessing mistreatment.
Hameed, Khan [56]	Pakistan	Cross-sectional	314	Protection from mistreatment	Supportive care was associated with reduced mistreatment and lower postpartum anxiety and depression.
Muhayimana, Kearns [38]	Rwanda	Qualitative	30	Respect, autonomy, privacy	Respectful care and autonomy were identified as key mechanisms for positive experiences.
Demirel, Kaya [50]	Turkey	Cross-sectional	686	Privacy, control	Single room environments were linked to greater privacy and control, improving experience.
Dahan and Goldberg [55]	Israel	Cross-sectional	788	Flow state, emotional presence	One-on-one support was linked to enhanced flow state and improved emotional presence.

Emotional security and relational trust formed another pathway [35,39,55,58]. Continuity of care models and doula support were often described as fostering supportive relationships that helped women feel safe, respected, and emotionally grounded during labour. Supportive care was also described as a buffer against mistreatment. Companions and doulas were reported to reduce the likelihood of disrespect, neglect, or coercion—factors that strongly shaped women’s overall experiences [56].

Most studies examined intrapartum care, with several collecting data postpartum to capture women’s reflections on their labour and birth experiences. These reflections provided insight into how women made sense of their childbirth experiences and how supportive care was linked to emotional well-being, perceived control, and overall satisfaction.

Taken together, the studies describe a set of psychological, perceptual, and relational mechanisms such as emotional safety, perceived control, trust, and narrative sense-making through which women interpret the role of supportive care during childbirth. These mechanisms help explain how women understand their experiences and why certain forms of support are consistently linked with more positive evaluations of childbirth.

### 3.4. Outcomes

Across the included studies, outcomes centred on how different forms of supportive care during childbirth shaped women’s childbirth experiences and overall satisfaction. The primary outcome examined across the review was maternal childbirth satisfaction, which was assessed through women’s perceptions of emotional support, respectful treatment, communication quality, companionship, and the general interpersonal environment of maternity care. The outcomes were examined mainly within the context of intrapartum care, although several studies collected postpartum data to understand how women interpreted their childbirth experiences.

Several studies (*n* = 13) focused specifically on intrapartum care, examining outcomes related to labour support, birth room interactions, birth environments, communication with providers, and the presence of companions [35,36,39,40,41,42,47,49,50,51,54,55,57]. These outcomes captured women’s real-time experiences of comfort, perceived safety, and support during labour.

A smaller group of studies (*n* = 6) examined postpartum outcomes, exploring women’s reflections on their birth experiences, retrospective satisfaction, and the lingering influence of supportive or unsupportive care after birth [37,38,46,53,56,59]. These outcomes provided insight into how women interpreted and evaluated their childbirth experiences in the weeks following birth.

Some studies (*n* = 6) assessed overall childbirth experience and satisfaction without restricting their analysis to a specific stage of labour or postpartum recovery [43,44,45,48,52,58]. These studies measured broad indicators such as emotional comfort, perceived respect, communication quality, and general satisfaction with facility-based care.

Across all studies, outcomes were categorised based on women’s reported experiences of emotional reassurance, perceived respect, autonomy, communication quality, and the presence or absence of supportive individuals. These outcomes were measured using validated satisfaction scales, structured questionnaires, labour support checklists, or postpartum interviews, depending on the study design.

#### 3.4.1. Maternity Care

Across the included studies, maternity care outcomes focused on women’s perceptions of emotional reassurance, respectful treatment, autonomy, communication, and the presence of supportive individuals within health facilities [45,48,52,58]. These outcomes captured women’s evaluations of the interpersonal and relational aspects of care that shaped their sense of dignity, comfort, and trust throughout childbirth [43,45,48]. Studies assessed these outcomes through women’s reports of how well providers communicated, whether they felt listened to, and the extent to which they were treated with empathy and respect [44,52,58]. Satisfaction within maternity care was closely linked to these interpersonal dimensions, with women consistently emphasising the importance of clear information sharing, emotional reassurance, and involvement in decision-making [43,45,48]. Together, these outcomes illustrate how supportive interpersonal care forms a central component of women’s overall evaluations of maternity care quality. Overall, the studies indicated that maternity care, whether delivered through interpersonal support, communication strategies, or structured models of care is closely linked to women’s satisfaction with childbirth. The results collectively highlight the importance of supportive, respectful, and responsive care as a cornerstone of positive maternity experiences.

#### 3.4.2. Childbirth Care

Childbirth represents the pivotal point of the maternity continuum, and several included studies concentrated specifically on support during labour [35,36,39,40,41,42,47,49,50,51,54,55,57]. Childbirth-specific outcomes focused on women’s intrapartum experiences, including emotional comfort, perceived safety, coping ability, and the presence or absence of continuous support. Across these intrapartum-focused studies, gaps in emotional, communicative, or structural support were associated with negative childbirth experiences, prompting examination of how different forms of supportive care shape the quality of childbirth [36,39,50]. These outcomes were central to understanding how supportive care was associated with women’s real-time experiences during labour. Much like the recognition of communication challenges in other maternity contexts, the studies in this review showed that shortcomings in provider–patient communication, empathic engagement, and continuous presence were linked to dissatisfaction and, in some cases, distress during labour [35,51,54].

Some authors focused on strengthening interpersonal communication during childbirth, showing that empathic provider behaviour and clear, respectful information sharing were associated with improved perceptions of safety and control among women [47,54,57]. Others emphasised the importance of companionship, noting that the presence of a partner, doula, or chosen support person during labour was linked to enhanced comfort, reduced fear, and more positive birth experiences [36,39,47]. In addition, studies examining labour room interactions highlighted that respectful treatment, autonomy, and confidentiality were central to women’s perceptions of supportive childbirth care [42,50,51,55]. Studies in this category examined how emotional reassurance, continuous presence, and clear communication contributed to women feeling calmer, more secure, and more in control during childbirth [36,40,41]. Outcomes such as perceived safety, emotional comfort, and the effectiveness of communication during labour were measured through labour support scales, observational tools, or immediate postpartum interviews. Across these studies, supportive intrapartum care, particularly the presence of a companion or midwife, assistance with positioning or breathing, and respectful communication was generally associated with more positive childbirth experiences and higher satisfaction [35,39,49].

Collectively, these studies reinforce the importance of supportive intrapartum environments. The ones in which communication is prioritised, emotional reassurance is consistently offered, and women feel accompanied rather than isolated during labour. These findings emphasise that childbirth care is not defined solely by clinical procedures but also by the quality of human interaction and the presence of supportive individuals throughout labour.

### 3.5. Postpartum Outcomes

Several studies collected data from women in the postpartum period, using surveys, interviews, or qualitative narratives to capture their reflections on labour and birth [37,38,46,53,56,59]. Although these studies did not examine postpartum care directly, they provided insight into how women interpreted their childbirth experiences after birth. Women frequently described feeling overlooked once clinical attention shifted toward the newborn, particularly in settings where communication was limited or support was inconsistent [37,59].

Postpartum reflections often highlighted how the quality of intrapartum support shaped women’s emotional responses after birth, including feelings of reassurance, coherence, or distress [38,53]. Some studies also explored postpartum emotional wellbeing, noting that women valued opportunities to discuss their birth experiences, receive clear information, and feel acknowledged by providers [56,59]. In addition, postpartum accounts showed that supportive companionship during labour contributed to women’s sense of safety and stability after birth, with Dubey, Sharma [46] demonstrating how positive intrapartum support shaped women’s early postpartum evaluations of their experience.

These accounts emphasised the importance of follow-up conversations, debriefing, or informal check-ins as ways to help women process their experiences and navigate early postpartum challenges. A number of authors described how mismatches between women’s expectations and their lived experiences contributed to feelings of vulnerability, particularly among those with limited resources or who experienced disrespect during labour [37,38].

Across these studies, postpartum reflections offered valuable insight into how women made sense of their childbirth experiences. These accounts highlighted the ongoing relevance of emotional support, clear communication, and partner involvement, not only during labour but also in shaping women’s interpretations of their experiences after birth. Collectively, this body of work illustrates how postpartum perspectives contribute to women’s overall evaluations of childbirth and their sense of wellbeing in the early weeks following delivery.

### 3.6. Methodological Approaches

The methodological approaches across the included studies varied according to their research aims but generally centred on examining women’s experiences of supportive care during childbirth. Most studies (*n* = 18) employed quantitative designs, including randomised controlled trials, quasi-experimental studies, cross-sectional surveys, and descriptive observational designs [40,41,42,43,44,45,46,47,48,49,50,51,52,53,54,55,56,57]. These studies primarily used structured questionnaires, validated satisfaction scales, labour support tools, or postpartum surveys to examine women’s perceptions of support, communication, autonomy, and overall childbirth satisfaction. Five studies (*n* = 5) adopted qualitative methodologies, using in-depth interviews or focus groups to explore women’s narratives and lived experiences of supportive or unsupportive care [35,36,37,38,39]. These studies offered rich, contextual insights into how emotional reassurance, respectful communication, and interpersonal interactions were understood and described by women during childbirth. A smaller number of studies (*n* = 2) used mixed-methods approaches, integrating qualitative insights with quantitative measures to provide a broader understanding of supportive care [58,59]. These designs enabled researchers to bring together numerical findings and narrative accounts, offering a more layered interpretation of women’s experiences.

Across qualitative and mixed-methods studies, data collection typically followed a two-phase structure, beginning with interviews or focus groups and followed by thematic analysis [35,36,37,38,39,58,59]. In contrast, quantitative studies generally used a single-phase design, collecting data through postpartum surveys, structured questionnaires, or observational assessments. In terms of data analysis, thematic analysis was the most frequently used approach in qualitative and mixed-method studies. Quantitative studies relied on descriptive statistics, regression models, and association testing to explore predictors of maternal satisfaction and the relationships between specific supportive care components and women’s reported experiences.

Despite differences in design, a common methodological pattern emerged. Initial phases generally involved data collection through interviews, questionnaires, or observational assessments of care interactions. Subsequent phases focused on analyzing these data to identify patterns in supportive care, communication quality, emotional reassurance, and their relationship to maternal satisfaction. In studies evaluating interventions such as empathic communication training, later phases included implementation, evaluation, and refinement of the intervention based on participant feedback.

Overall, the methodological approaches across the included studies offered a strong foundation for understanding how supportive care is experienced and evaluated by women during childbirth, capturing both measurable outcomes and the nuanced, interpretive dimensions of maternity care.

## 4. Discussion

This review shows that women’s childbirth experiences and satisfaction are shaped less by clinical procedures and more by the interpersonal and emotional conditions under which care is delivered. Across different settings and study designs, supportive care generally emerged as a central element of positive maternity experiences [35,36,37,39]. Emotional reassurance, respectful communication, clear information, and the presence of trusted companions were repeatedly described as essential to women’s sense of safety, dignity, and control during childbirth [51,54,57,58].

Although the included studies varied in focus, some examined overall childbirth experience while others concentrated on intrapartum processes or postpartum reflections, the pattern of findings was consistent. Supportive care functioned as a relational framework that helped women manage uncertainty, reduce fear, and make meaning of their childbirth experiences [36,40,41,43,52]. Even when satisfaction was not the primary outcome, studies highlighted emotional and psychological pathways through which support contributed to wellbeing, including increased confidence, and greater trust in providers [46,50,55].

The review also reinforces that supportive care can protect women in contexts where disrespect or mistreatment are concerns. Companions, doulas, and continuity-of-care models often acted as buffers against neglect or coercion, helping women feel informed, protected, and emotionally secure [35,37,56]. In contrast, gaps in communication or emotional support were frequently linked to fear, trauma, and negative interpretations of childbirth [38,51,53]. These patterns show that supportive care is not simply an added benefit but a key determinant of how women interpret and evaluate their maternity experiences.

Postpartum reflections in the included studies reinforced this interpretation. Studies that collected data after birth showed that women continued to make sense of their experiences through the lens of the support they received during labour [38,59]. Supportive communication and emotional presence helped women construct coherent narratives about childbirth, even when complications occurred [37,56]. In contrast, unsupportive or disrespectful care often left women with unresolved emotions and difficulty integrating their experiences [48,49,51]. These findings highlight the temporal dimension of supportive care: its influence extends beyond the moment of birth into women’s later emotional processing and meaning-making.

Taken together, the evidence suggests that supportive care generally operates through interconnected emotional, relational, and communicative mechanisms. Lower fear and anxiety, enhanced autonomy, clearer understanding, and stronger relational trust could all contribute to more positive childbirth experiences [40,45,46,47]. Satisfaction could therefore reflects cumulative interpersonal experiences rather than isolated clinical events. This interpretation aligns with broader maternity care literature emphasising the importance of respectful, person-centred care.

From a practice perspective, these findings highlight the need to embed supportive care within routine maternity services. Training that prioritises emotional responsiveness, respectful communication, and shared decision-making can improve women’s experiences without requiring major technological investment. Policies that allow chosen companions and promote continuity of interpersonal care can also strengthen women’s sense of safety and trust, especially in resource-constrained settings. The methodological appraisal indicated that most included studies met the majority of the relevant MMAT criteria. As a result, the appraisal informed the interpretation of the findings rather than serving as a basis for differential weighting across studies.

Overall, this review identifies supportive care as a core component of high-quality maternity care. Emotional, relational, and communicative dimensions of support consistently shaped women’s interpretations of childbirth and their overall satisfaction. Strengthening these aspects of care may help maternity services better align with women’s needs and expectations, ultimately contributing to more positive and meaningful childbirth experiences.

### 4.1. Strengths and Limitations

A key strength of this review is its inclusion of diverse study designs, including quantitative, qualitative, and mixed-methods research. This range allowed for a comprehensive understanding of supportive care across the maternity continuum. By focusing on studies published between 2020 and 2025, the review also draws on evidence that reflects recent maternity care practices. While the studies did not all examine the same aspects of supportive care, many addressed evolving approaches to communication, doula support, and companionship. Integrating psychosocial, experiential, and relational dimensions of care further strengthened the interpretive depth of the review.

This review has several limitations. First, restricting the search to English-language publications may have introduced language bias by excluding relevant studies from non-English-speaking contexts, which could limit the global representativeness of the findings. Second, variation in how supportive care was defined and measured across studies made direct comparison difficult. The use of narrative synthesis, although appropriate for heterogeneous evidence, did not allow for statistical pooling or the calculation of effect sizes. As a result, the strength of associations could not be quantified. Heterogeneity in study designs, measurement tools, and definitions of supportive care also limited comparability across studies. In addition, most included studies were conducted in facility-based settings, which may reduce the transferability of findings to community or home-birth contexts. Furthermore, the predominance of cross-sectional designs limits conclusions about the long-term effects of supportive care on women’s wellbeing and trust in the health system. Finally, because this review employed a narrative synthesis of heterogeneous qualitative, quantitative, and mixed-methods studies rather than a meta-analysis, a formal assessment of publication bias (e.g., funnel plots or statistical tests) was not undertaken.

Future research could examine how supportive care is implemented and sustained within resource-constrained health systems and explore the mechanisms through which it influences women’s experiences across different sociocultural contexts. Further studies using standardized outcome measures and longitudinal designs may help improve understanding of the longer-term associations between supportive care and women’s childbirth experiences and related outcomes.

### 4.2. Implications for Practice

The findings of this review have implications for individual clinical practice, maternity unit organisation, and health policy. Although the included studies varied in design, setting, and methodological quality, the evidence generally suggests that supportive care is associated with more positive childbirth experiences and higher maternal satisfaction.

Implications for individual clinical practice. The findings highlight the importance of integrating supportive care behaviours into routine intrapartum care. Emotional reassurance, respectful and empathic communication, continuous presence, and support for informed decision-making were generally associated with more positive childbirth experiences across the included studies. Because these practices require few additional clinical resources, they may be feasibly incorporated into routine maternity care across diverse healthcare settings. Incorporating communication and supportive care skills into continuing professional development programmes for midwives, nurses, and obstetric staff may further strengthen women’s experiences of care.

Implications for maternity unit organisation. The review underscores the value of organisational practices that facilitate supportive care throughout labour and childbirth. Studies reporting on labour companionship indicated that women often felt more secure, informed, and emotionally supported when accompanied by a trusted individual. Maternity units may therefore consider implementing clear protocols that support the presence of a woman’s chosen birth companion, where appropriate. The findings also suggest that structured postpartum debriefing, follow-up communication, and accessible informational resources may help women process their childbirth experiences and enhance emotional wellbeing after birth.

Implications for health policy. At the policy level, the findings suggest continued efforts to embed supportive care within standards for quality maternity services. Policies that promote respectful maternity care, support effective provider communication, facilitate informed decision-making, and enable labour companionship may strengthen woman-centred maternity care. However, given the heterogeneity of the included studies and the absence of meta-analysis, these recommendations should be interpreted cautiously and adapted to local healthcare contexts and available resources.

## 5. Conclusions

This systematic review reveals that women’s satisfaction with childbirth care is closely associated with supportive care, in addition to clinical outcomes. Across varied contexts and methodologies, supportive care was generally associated with how childbirth was experienced, interpreted, and remembered in the included studies. Emotional reassurance, respectful communication, companion presence, and autonomy-supporting practices emerged as dominant components that were linked to more meaningful and affirming experiences for women.

Notably, the review identified several psychosocial pathways through which supportive care shaped childbirth experiences. Supportive care was generally associated with emotional safety, perceived control, mental wellbeing, and narrative sense-making in the included studies. These factors were also linked to women’s immediate experiences of labour and later reflections on childbirth. Satisfaction, therefore, reflects cumulative and interpretive experiences shaped by the quality of interpersonal engagement across labour and the early postpartum period.

These findings have important implications for maternity care systems. The findings suggest that supportive care is an important component of woman-centred maternity care and may contribute to improved childbirth experiences. However, the strength of evidence varies across study designs, settings, and supportive care interventions.

## Figures and Tables

**Table 1:** Inclusion and exclusion Criteria.

Description	Inclusion Criteria	Exclusion Criteria
**Population**	Women receiving facility-based intrapartum or postpartum care; birth companions, doulas, or healthcare providers who provided supportive care during childbirth	Women receiving care outside health facilities (home births), or non-pregnant women
**Focus**	Supportive dimensions of maternity care	Studies focusing solely on clinical procedures or technical care without any interpersonal/supportive aspect
**Outcome**	Maternal satisfaction or experiential outcomes	Studies not reporting maternal satisfaction or experiential outcomes
**Study design**	Quantitative, qualitative, mixed-methods	Case reports, commentaries, reviews, editorials, protocols
**Time frame**	2020–2025	Studies published before 2020 or after 2025

**Table 2:** Complete Search Strategy for Each Database.

Database	Search Strategy	Filters/Limits Applied
MEDLINE (PubMed)	(“birth” OR “labour” OR “labor” OR “delivery” OR “childbirth” OR “maternity care”) AND (“support *” OR “supportive care” OR “labour support” OR “continuous support” OR “birth support” OR “birth companion *” OR “birth companionship” OR “companion *” OR “companion support” OR “doula *” OR “emotional support” OR “respectful maternity care” OR “person-centred maternity care” OR “person centered maternity care”) AND (“experience *” OR “birth experience” OR “childbirth experience” OR “satisfaction” OR “maternal satisfaction” OR “women’s experience *” OR “patient experience”)	Language: English language; Year: 2020–2025; Document Type: Articles. Controlled vocabulary (MeSH terms) and free-text keywords were used where applicable.
Web of Science	TS = (“birth” OR “labour” OR “labor” OR “delivery” OR “childbirth” OR “maternity care”) AND TS = (“support *” OR “supportive care” OR “labour support” OR “continuous support” OR “birth support” OR “birth companion *” OR “birth companionship” OR “companion *” OR “companion support” OR “doula *” OR “emotional support” OR “respectful maternity care” OR “person-centred maternity care” OR “person centered maternity care”) AND TS = (“experience *” OR “birth experience” OR “childbirth experience” OR “satisfaction” OR “maternal satisfaction” OR “women’s experience *” OR “patient experience”)	Language: English language; Year: 2020–2025; Document Type: Articles.
CINAHL (EBSCOhost)	(“birth” OR “labour” OR “labor” OR “delivery” OR “childbirth” OR “maternity care”) AND (“support *” OR “supportive care” OR “labour support” OR “continuous support” OR “birth support” OR “birth companion *” OR “birth companionship” OR “companion *” OR “companion support” OR “doula *” OR “emotional support” OR “respectful maternity care” OR “person-centred maternity care” OR “person centered maternity care”) AND (“experience *” OR “birth experience” OR “childbirth experience” OR “satisfaction” OR “maternal satisfaction” OR “women’s experience *” OR “patient experience”)	Language: English language; Year: 2020–2025; Document Type: Articles. CINAHL subject headings were considered where available.
Scopus	TITLE-ABS-KEY (“birth” OR “labour” OR “labor” OR “delivery” OR “childbirth” OR “maternity care”) AND TITLE-ABS-KEY (“support *” OR “supportive care” OR “labour support” OR “continuous support” OR “birth support” OR “birth companion *” OR “birth companionship” OR “companion *” OR “companion support” OR “doula *” OR “emotional support” OR “respectful maternity care” OR “person-centred maternity care” OR “person centered maternity care”) AND TITLE-ABS-KEY (“experience *” OR “birth experience” OR “childbirth experience” OR “satisfaction” OR “maternal satisfaction” OR “women’s experience *” OR “patient experience”)	Language: English language; Year: 2020–2025; Document Type: Articles.
PsycINFO	(“birth” OR “labour” OR “labor” OR “delivery” OR “childbirth” OR “maternity care”) AND (“support *” OR “supportive care” OR “labour support” OR “continuous support” OR “birth support” OR “birth companion *” OR “birth companionship” OR “companion *” OR “companion support” OR “doula *” OR “emotional support” OR “respectful maternity care” OR “person-centred maternity care” OR “person centered maternity care”) AND (“experience *” OR “birth experience” OR “childbirth experience” OR “satisfaction” OR “maternal satisfaction” OR “women’s experience *” OR “patient experience”)	Language: English language; Year: 2020–2025; Document Type: Articles. APA Thesaurus terms were considered where applicable.

The asterisk (*) denotes truncation and retrieves variations of a word root (e.g., support * retrieves support, supports, supportive, and supporting).

**Table 3:** MMAT Quality Appraisal of Included Studies (2020–2025).

MMAT Methodological Quality Criteria for Qualitative Study Author and Year of Publication												
	Scheenen, Timmers [35]		Wanyenze, Byamugisha [36]			Hagaman, Rodriguez [37]		Muhayimana, Kearns [38]			Summerton, Mtileni [39]	
1.1 Is the qualitative approach appropriate to answer the research question?	Y		Y			Y		Y			Y	
1.2 Are the qualitative data collection methods adequate to address the research question?	Y		Y			Y		Y			Y	
1.3 Are the findings adequately derived from the data?	Y		Y			Y		Y			Y	
1.4 Is the interpretation of results sufficiently substantiated by data?	Y		Y			Y		Y			Y	
1.5 Is there coherence between qualitative data sources, collection, analysis and interpretation?	Y		Y			Y		Y			Y	
**Comments**	Clear data collection and analysis; strong reflexivity		Credible analysis; strong thematic development			Strong thematic saturation and credibility		Strong reflexivity and credibility			Strong methodological transparency	
**MMAT Methodological Quality Criteria for Quantitative randomized controlled trials** **Author and Year of Publication**												
	**Stjernholm, Charvalho [40]**			**Balcik Colak, Akin [41]**			**Schytt, Wahlberg [42]**			**Akçay and Alan [43]**		
2.1. Is randomization appropriately performed?	Y			Y			Y			Y		
2.2. Are the groups comparable at baseline?	Y			Y			Y			Y		
2.3. Are there complete outcome data?	Y			Y			Y			Y		
2.4. Are outcome assessors blinded to the intervention provided?	N			N			N			Y		
2.5 Did the participants adhere to the assigned intervention?	Y			Y			Y			Y		
**Comments**	Randomisation appropriate; blinding not fully reported			Randomisation adequate; some risk of performance bias			Appropriate design; limited blinding			Strong randomisation and outcome measurement		
**MMAT Methodological Quality Criteria for Quantitative non-randomized** **Author and Year of Publication**												
	**Jeong, Kim [44]**			**Nakphong, Afulani [45]**			**Dubey, Sharma [46]**			**Shahbazi Sighaldeh, Azadpour [47]**		
3.1. Are the participants representative of the target population?	Y			Y			Y			Y		
3.2. Are measurements appropriate regarding both the outcome and intervention (or exposure)?	Y			Y			Y			Y		
3.3. Are there complete outcome data?	Y			Y			Y			Y		
3.4. Are the confounders accounted for in the design and analysis?	N			Y			N			N		
3.5. During the study period, is the intervention administered (or exposure occurred) as intended?	Y			Y			Y			Y		
**Comments**	Confounding not fully controlled			Robust analysis with adjustment for confounders			Confounding not fully controlled			Limited control of confounding		
**MMAT Methodological Quality Criteria for Quantitative descriptive** **Author and Year of Publication**												
	**Liu, Li [48]**	**Alwahaibi, Al-Julandani [49]**	**Demirel, Kaya [50]**		**Van der Pijl, Verhoeven [51]**	**Alwahaibi, Alajaimi [52]**	**Li, Xiao [53]**	**Van der Pijl, Kasperink [54]**	**Dahan and Goldberg [55]**		**Hameed, Khan [56]**	**Kiti, Prata [57]**
4.1. Is the sampling strategy relevant to address the research question?	Y	Y	Y		Y	Y	Y	Y	Y		Y	Y
4.2. Is the sample representative of the target population?	Y	Y	N		Y	Y	Y	Y	Y		Y	Y
4.3. Are the measurements appropriate?	Y	Y	Y		Y	Y	Y	Y	Y		Y	Y
4.4. Is the risk of nonresponse bias low?	Y	Y	Y		Y	Y	Y	Y	Y		Y	Y
4.5. Is the statistical analysis appropriate to answer the research question?	Y	Y	Y		Y	Y	Y	Y	Y		Y	Y
**Comments**	Sampling strategy appropriate; measurements valid	Appropriate sampling and statistical analysis	Valid sampling and measurement tools used but Minor sampling limitations		Excellent sampling and measurement validity	Strong internal consistency and analysis	Validated instruments and appropriate analysis	Clear sampling and robust statistical methods	Valid measures and strong statistical analysis		Appropriate design and statistical rigor	Strong adjusted analysis and validity of measures
**MMAT Methodological Quality Criteria for Mixed methods** **Author and Year of Publication**												
	**Purandare, Ådahl [58]**						**[59]**					
5.1. Is there an adequate rationale for using a mixed methods design to address the research question?	Y						Y					
5.2. Are the different components of the study effectively integrated to answer the research question?	Y						Y					
5.3. Are the outputs of the integration of qualitative and quantitative components adequately interpreted?	Y						Y					
5.4. Are divergences and inconsistencies between quantitative and qualitative results adequately addressed?	Y						Y					
5.5. Do the different components of the study adhere to the quality criteria of each tradition of the methods involved?	Y						Y					
**Comments**	Integration of qualitative and quantitative components clearly justified						Strong integration of qualitative and quantitative components					

Legend: Y = Yes, N = No, CT = Can’t tell response. Bold text indicates table headings and section headings.

**Table 4:** Summary of Included Study Characteristics (2020–2025).

No.	Author	Title and Year	Country	Design	Sample Size	Participants	Aim	Support Type	Key Findings/Outcomes
1	Stjernholm, Charvalho [40]	Continuous Support Promotes Obstetric Labor Progress and Vaginal Delivery in Primiparous Women—A Randomized Controlled Study (2021)	Sweden	RCT	59	Primiparous women	To test continuous support vs. standard care	Continuous emotional and physical support	Continuous support reduced anxiety, improved coping, and enhanced satisfaction with birth
2	Balcik Colak, Akin [41]	Effects of labor support on pregnant women’s childbirth comfort, satisfaction and postpartum comfort levels: a randomized controlled trial (2025)	Turkey	RCT	104	Primigravidae	To evaluate companion-led support	Breathing guidance, reassurance	Reduced fear, improved labour coping, and increased maternal satisfaction
3	Liu, Li [48]	Women’s experience and satisfaction with midwife-led maternity care: a cross sectional survey in China (2021)	China	Cross-sectional	4192	Laboring women who had vaginal births	To examine labour practices	Free positioning, epidural use	Supportive practices were associated with reduced interventions and improved maternal comfort
4	Scheenen, Timmers [35]	Women’s experiences with continuous care during labor by maternity care assistants in The Netherlands: a qualitative study (2025)	Netherlands	Qualitative	14	Women in continuous care trial	To explore experiences with maternity care assistants	Continuous maternity care	Women valued continuity, emotional presence, and personalised support; improved trust and reduced anxiety
5	Wanyenze, Byamugisha [36]	A qualitative exploratory interview study on birth companion support actions for women during childbirth (2022)	Uganda	Qualitative	10	Women with companions	To explore women’s perceptions of companion support	Emotional and physical support	Companions reduced fear, provided reassurance, and improved women’s sense of dignity and comfort
6	Alwahaibi, Al-Julandani [49]	The role and effect of companions during childbirth in Oman (2024)	Oman	Cross-sectional	214	Birth companions	To assess companions’ support roles	Emotional, physical, communication support	Companions enhanced women’s comfort, reduced stress, and improved communication with staff
7	Schytt, Wahlberg [42]	Community-based bilingual doula support during labour and birth to improve migrant women’s intrapartum care experiences and emotional well-being–Findings from a randomised controlled trial in Stockholm, Sweden [NCT03461640] (2022)	Sweden	RCT	164	Migrant women	To test effects of professional doula support	Continuous labour support	Doula support reduced childbirth fear, increased satisfaction, and improved emotional security compared to standard care
8	Purandare, Ådahl [58]	Migrant women’s experiences of community-based doula support during labor and childbirth in Sweden. A mixed methods study (2024)	Sweden	Mixed-methods	82 + 12 qualitative	Migrant women	To explore migrant women’s experiences with community-based doulas	Emotional, informational, advocacy support	Doulas improved communication, reduced fear, enhanced trust in providers, and increased perceived safety and cultural understanding
9	Jeong, Kim [44]	Effect of a continuity of midwifery care model that used a respectful maternal care framework in Korea: a non-randomized study (2025)	South Korea	Non-randomised	63	Pregnant women	To evaluate continuity midwifery care	Team-Mamas model	Improved childbirth confidence, reduced anxiety, and strengthened continuity of care relationships
10	Demirel, Kaya [50]	The Effects of the Number of Pregnant in the Labor Room on Perception of Support, Fear of Childbirth and Satisfaction (2022)	Turkey	Cross-sectional	686	Postnatal women	To examine how labour room type affects care experience	Single vs. shared labour rooms	Shared rooms were associated with reduced privacy, lower autonomy, and poorer perceptions of respectful care
11	Nakphong, Afulani [45]	Integrating support persons into maternity care and associations with quality of care: a postpartum survey of mothers and support persons in Kenya (2024)	Kenya	Cross-sectional	1138 + 606	Women + support persons	To evaluate Person-Centred Integration of Support Persons (PC-ISP) structured involvement	Support persons integrated into communication	Increased autonomy, improved shared decision-making, and enhanced respectful care
12	Hagaman, Rodriguez [37]	“Even though they insult us, the delivery they give us is the greatest thing”: a qualitative study contextualizing women’s experiences with facility-based maternal health care in Ethiopia (2022)	Ethiopia	Qualitative	41	Postpartum women	To explore respectful care experiences	Labor support	Women reported communication gaps, delays, and mistreatment; supportive providers improved trust, comfort and satisfaction outcomes.
13	Van der Pijl, Verhoeven [51]	Disrespect and abuse during labour and birth amongst women in the Netherlands (2022)	Netherlands	National Cross-sectional survey	12,239	Postnatal women	To assess respectful care and autonomy	Experience-of-care survey	Identified gaps in communication, autonomy, and emotional support; disrespect and lack of involvement were common
14	Alwahaibi, Alajaimi [52]	Women’s preferences, impacts, and satisfaction with companion support during labour and delivery experiences in Oman. (2025)	Oman	Cross-sectional	444 + 444	Women + companions	To examine support across admission–discharge	Family companion support	High levels of support were associated with improved satisfaction, emotional comfort, and perceived safety
15	Li, Xiao [53]	Partner involvement and emotional support gaps as predictors of birth trauma symptoms: a multi-center cross-sectional study of 230 women at 42 days (2025)	China	Cross-sectional	230	Postnatal women	To assess partner involvement	Emotional and informational support	Low partner involvement was linked to poorer emotional well-being and reduced satisfaction
16	Muhayimana, Kearns [38]	Experiences and perceptions of respectful maternity care among mothers during childbirth in health facilities of Eastern province of Rwanda: An appreciative inquiry (2025)	Rwanda	Qualitative	30	Women reporting RMC	To explore respectful maternity care	Compassionate care, autonomy	Positive experiences tied to respectful care improved emotional well-being, trust, and perceived dignity
17	Van der Pijl, Kasperink [54]	Client-care provider interaction during labour and birth as experienced by women: Respect, communication, confidentiality and autonomy (2021)	Netherlands	Cross-sectional	767	Postnatal women	To assess respectful care	Communication, confidentiality	Identified deficits in autonomy, communication, and emotional support
18	Dahan and Goldberg [55]	Birthing With Others: Exploring the Efficacy of One-On-One Professional Support on Physiological Birth and Flow State (2025)	Israel	Cross-sectional	788	Women with childbirth experience	To examine private midwife/doula support	One-on-one continuous support	Support improved physiological birth outcomes, reduced interventions, and enhanced “flow state”
19	Dubey, Sharma [46]	Impact of Birth Companionship on Maternal and Fetal Outcomes in Primigravida Women in a Government Tertiary Care Center (2023)	India	Observational	150	Primigravida women	To test effects of birth companionship	Emotional support, massage, mobility	Companionship shortened labour duration, reduced anxiety, and improved breastfeeding initiation
20	Ihudiebube-Splendor, Enwereji-Emeka [59]	Childbirth experiences and satisfaction with birth among women in selected Nigerian healthcare facilities during COVID-19 pandemic: a mixed method (2024)	Nigeria	Mixed-methods	280 + 15	Postpartum women	To explore support during COVID-19	Partner, midwife, family support	COVID-19 restrictions reduced support, increasing fear and reducing satisfaction
21	Akçay and Alan [43]	The effect of empathic communication-based training on women’s positive birth perception, awareness, and birth experience: a randomized controlled trial (2025)	Turkey	RCT	72	Primiparous women	To test empathic communication training	Empathic communication	Improved birth perception, reduced fear, and enhanced emotional readiness
22	Summerton, Mtileni [39]	Experiences and perceptions of birth companions supporting women in labour at a District Hospital in Limpopo, South Africa (2021)	South Africa	Qualitative	73	Birth companions	To document companions’ experiences	Emotional and physical support	Companions reported overwhelmingly positive experiences; some noted disrespect from staff
23	Hameed, Khan [56]	The role of birth companionship in women’s experiences of mistreatment during childbirth and postpartum anxiety and depression: An analysis of a cross-sectional survey (2025)	Pakistan	Cross-sectional	314	Postnatal women	To examine companionship, mistreatment and mental health	Emotional, informational, instrumental support	High support from companions reduced mistreatment and postpartum anxiety
24	Shahbazi Sighaldeh, Azadpour [47]	Comparison of maternal outcomes in caring by Doula, trained lay companion and routine midwifery care (2023)	Iran	Quasi-experimental	150	Low-risk pregnant women	To compare doula vs. lay vs. routine care	Doula and trained companion support	Doula support reduced anxiety, shortened labour, and improved satisfaction
25	Kiti, Prata [57]	Continuous Labor Support and Person-Centered Maternity Care: A Cross-Sectional Study with Women in Rural Kenya (2022)	Kenya	Cross-sectional	865	Recently delivered women	To assess continuous labour support	Lay companion support	Higher Person-Centred Maternity Care (PCMC) scores (dignity, respect, communication) among women with companions

## Data Availability

No new datasets were generated during this study. All data analysed were obtained from published studies cited in this article.

## Supplementary Materials

The following supporting information can be downloaded at: https://www.mdpi.com/article/10.3390/healthcare14142041/s1, The PRISMA 2020 checklist supporting this systematic review is available as Supplementary File S1 [60].

## Acronyms

CBD	Community-Based Doula
CI	Confidence Interval
COVID-19	Coronavirus Disease 2019
GBD	Global Burden of Disease
MMAT	Mixed Methods Appraisal Tool
PC-ISP	Person-Centred Integration of Support Persons
PCMC	Person-Centred Maternity Care
PRISMA	Preferred Reporting Items for Systematic Reviews and Meta-Analyses
PRISMA-S	Preferred Reporting Items for Systematic Reviews and Meta-Analyses Search Extension
RCT	Randomized Controlled Trial
RIS	Research Information Systems (bibliographic exchange format)
RQ	Research Question
WHO	World Health Organization
