# Supplementary material for: How Supportive Care Shapes Women’s Childbirth Satisfaction: A Systematic Review
Source: Healthcare (Basel). 2026 Jul 8;14(14):2041. doi: 10.3390/healthcare14142041 (PMC13409745; doi:10.3390/healthcare14142041)
Supplement: Supplementary file 1 [file healthcare-14-02041-s001.zip › healthcare-4364107-supplementary File S1.pdf]

PRISMA 2020 Checklist

Section and Topic	Item #	Checklist item	Location where item is reported
**TITLE**			
Title	1	Identify the report as a systematic review.	*How Supportive Care Shapes Women's Childbirth Satisfaction: A Systematic Review*
**ABSTRACT**			
Abstract	2	See the PRISMA 2020 for Abstracts checklist.	**Abstract**<br>**Background:**<br>Despite strong clinical safety standards, many women continue to report negative or unsatisfactory childbirth experiences. Supportive care has gained attention as an important component of quality maternity services, yet evidence on its relationship with women's satisfaction remains limited. **Objective:** This systematic review examined recent research on supportive care during childbirth and its association with women's satisfaction and childbirth experiences. **Methods:** A systematic search of PubMed/MEDLINE, Web of Science, CINAHL, PsycINFO, and Scopus was conducted for peer-reviewed studies published between 2020 and 2025. Quantitative, qualitative, and mixed-methods studies examining supportive care during childbirth and maternal satisfaction were included. Twenty-five studies met the inclusion criteria. Data were synthesised narratively and supported by bibliometric co-occurrence mapping using VOSviewer to identify dominant thematic clusters. Included studies comprised randomised controlled trials, cross-sectional studies, quasi-experimental studies, qualitative interviews, and mixed-methods designs. **Results:** Supportive care was consistently associated with higher maternal satisfaction and more positive childbirth experiences. Common forms of support included emotional support, respectful communication, companionship, informational support, and autonomy-supportive care. Psychosocial factors such as emotional safety, perceived control, and mental wellbeing were reported as being associated with women's experiences.<br>**Conclusion:** Supportive care is an important component in the literature on women's childbirth satisfaction. Emotional and relational aspects of care were frequently highlighted across studies. Integrating supportive practices into routine maternity care may contribute to more woman-centred approaches while maintaining clinical safety.
**INTRODUCTION**			
Rationale	3	Describe the rationale for the review in the context of existing knowledge.	The rationale for this review is grounded in the growing recognition that supportive care is a fundamental component of high-quality intrapartum maternity services. Supportive care includes emotional support, informational support, physical comfort measures, continuous presence, advocacy, and respectful communication provided to women during facility-based childbirth. These elements are widely endorsed by global health bodies and are closely linked to women's perceptions of dignity, safety, and satisfaction during childbirth. In spite of this recognition, the existing evidence base is fragmented and inconsistent, with studies varying in context, definitions, and focus. This lack of synthesis limits the ability of clinicians, policymakers, and maternity care systems to understand which supportive care practices strongly influence maternal satisfaction.<br>The need for an updated review is further justified by the rapid evolution of maternity care practices between 2020 and 2025, a period shaped by evolving maternity care practices, including changes driven by global health system pressures. These shifts may have altered how supportive care is delivered and experienced, making it essential to reassess the contemporary evidence. Therefore, this review is needed to consolidate recent findings, identify which forms of supportive care are most influential, and clarify the mechanisms through which supportive care shapes women's childbirth satisfaction.
Objectives	4	Provide an explicit statement of the objective(s) or question(s) the review addresses.	1. How does supportive care during childbirth influence maternal satisfaction?<br>2. Which forms of supportive care are most strongly associated with satisfaction?<br>3. What pathways or mechanisms link supportive care to positive childbirth experiences?
**METHODS**			
Eligibility criteria	5	Specify the inclusion and exclusion criteria for the review and how studies were	Inclusion criteria:<br>Studies involving women who gave birth in health facilities, reporting maternal satisfaction or childbirth experience in relation to supportive care. Eligible designs included RCTs, non-randomised quantitative studies, qualitative studies, and mixed-methods studies.

Section and Topic	Item #	Checklist item	Location where item is reported
		grouped for the syntheses.	Supportive care included emotional, informational, physical comfort, continuous presence, advocacy, and respectful maternity care practices.<br>Exclusion criteria:<br>Studies on home births, antenatal or postnatal care only, studies without satisfaction/experience outcomes, studies evaluating only clinical/medical interventions, non-English publications, and studies published before 2020.<br>How studies were grouped for synthesis:<br>Because no formal meta-analysis was planned, studies were grouped narratively according to:<br>Type of supportive care (emotional, informational, physical, continuous presence, advocacy)<br>Study design (quantitative, qualitative, mixed-methods)<br>Reported outcomes (maternal satisfaction, supportive care components, mechanisms linking support to experience)<br>This grouping allowed a structured narrative synthesis of findings.
Information sources	6	Specify all databases, registers, websites, organisations, reference lists and other sources searched or consulted to identify studies. Specify the date when each source was last searched or consulted.	The following sources were searched to identify eligible studies:<br>Databases: CINAHL, MEDLINE, PsycINFO, PubMed, Scopus, Web of Science<br>Other sources: Forward citation searching ("snowballing"), backward reference list checking<br>Registers/websites/organisations: None reported<br>Unpublished studies: Not searched (only published studies were sought)<br>Date last searched:<br>All databases and sources were searched for studies published from 1 January 2020 to 31 December 2025, as stated in the protocol. The formal search was completed during the review's formal searching/study identification stage, which is marked as started in the PROSPERO record.
Search strategy	7	Present the full search strategies for all databases, registers and websites, including any filters and limits used.	The following search strategies were used across all databases.<br>The same core Boolean structure was applied, with database-specific syntax where required.<br>All searches were limited to English-language, peer-reviewed articles published from 2020 to 2025.<br>1. MEDLINE (PubMed)-search string<br>("birth" OR "labour" OR "labor" OR "delivery" OR "childbirth" OR "maternity care")<br>AND<br>("support*" OR "supportive care" OR "labour support" OR "continuous support" OR "birth support" OR "birth companion*" OR "birth companionship" OR "companion*" OR "companion support" OR "doula*" OR "emotional support" OR "respectful maternity care" OR "person-centred maternity care" OR "person centered maternity care")<br>AND<br>("experience*" OR "birth experience" OR "childbirth experience" OR "satisfaction" OR "maternal satisfaction" OR "women's experience*" OR "patient experience")<br>Filters: Language: English language; Year: 2020–2025; Document Type: Articles. Controlled vocabulary (MeSH terms) and free-text keywords were used where applicable.<br>2. Web of Science (Core Collection)-search string<br>TS= ("birth" OR "labour" OR "labor" OR "delivery" OR "childbirth" OR "maternity care")<br>AND TS=("support*" OR "supportive care" OR "labour support" OR "continuous support" OR "birth support" OR "birth companion*" OR "birth companionship" OR "companion*" OR "companion support" OR "doula*" OR "emotional support" OR "respectful maternity care" OR "person-centred maternity care" OR "person centered maternity care") AND TS=("experience*" OR "birth experience" OR

Section and Topic	Item #	Checklist item	Location where item is reported
			"childbirth experience" OR "satisfaction" OR "maternal satisfaction" OR "women's experience*" OR "patient experience")<br>Filters: Language: English language; Year: 2020–2025; Document Type: Articles.<br>3. CINAHL (EBSCO)-search string<br>("birth" OR "labour" OR "labor" OR "delivery" OR "childbirth" OR "maternity care")<br>AND<br>("support*" OR "supportive care" OR "labour support" OR "continuous support" OR "birth support" OR "birth companion*" OR "birth companionship" OR "companion*" OR "companion support" OR "doula*" OR "emotional support" OR "respectful maternity care" OR "person-centred maternity care" OR "person centered maternity care")<br>AND<br>("experience*" OR "birth experience" OR "childbirth experience" OR "satisfaction" OR "maternal satisfaction" OR "women's experience*" OR "patient experience")<br>Filters: English language; Year: 2020–2025; Document Type: Articles. CINAHL subject headings were considered where available.<br>4. Scopus-search string<br>TITLE-ABS-KEY ("birth" OR "labour" OR "labor" OR "delivery" OR "childbirth" OR "maternity care")<br>AND TITLE-ABS-KEY ("support*" OR "supportive care" OR "labour support" OR "continuous support" OR "birth support" OR "birth companion*" OR "birth companionship" OR "companion*" OR "companion support" OR "doula*" OR "emotional support" OR "respectful maternity care" OR "person-centred maternity care" OR "person centered maternity care")<br>AND TITLE-ABS-KEY("experience*" OR "birth experience" OR "childbirth experience" OR "satisfaction" OR "maternal satisfaction" OR "women's experience*" OR "patient experience")<br>Filters: Language: English language; Year: 2020–2025; Document Type: Articles.<br>5. PsycINFO- search string<br>("birth" OR "labour" OR "labor" OR "delivery" OR "childbirth" OR "maternity care")<br>AND ("support*" OR "supportive care" OR "labour support" OR "continuous support" OR "birth support" OR "birth companion*" OR "birth companionship" OR "companion*" OR "companion support" OR "doula*" OR "emotional support" OR "respectful maternity care" OR "person-centred maternity care" OR "person centered maternity care")<br>AND ("experience*" OR "birth experience" OR "childbirth experience" OR "satisfaction" OR "maternal satisfaction" OR "women's experience*" OR "patient experience")<br>Filters: Language: English language; Year: 2020–2025; Document Type: Articles. APA Thesaurus terms were considered where applicable.<br>Other Sources<br>Reference lists of included studies were screened manually.<br>Forward citation searching was conducted to identify additional studies.
Selection process	8	Specify the methods used to decide whether a study met the inclusion criteria of the review, including how many reviewers screened each record and each report retrieved, whether	Screening was conducted in two stages:<br>Title and abstract screening, and Full-text screening.<br>Two reviewers independently screened all records and full-text reports to determine eligibility according to the predefined inclusion criteria. Disagreements were resolved through discussion, and when necessary, by consulting a third reviewer. No automation tools were used at any stage of the screening process. Reference lists of included studies were also screened manually.

Section and Topic	Item #	Checklist item	Location where item is reported
		they worked independently, and if applicable, details of automation tools used in the process.	
Data collection process	9	Specify the methods used to collect data from reports, including how many reviewers collected data from each report, whether they worked independently, any processes for obtaining or confirming data from study investigators, and if applicable, details of automation tools used in the process.	Data were extracted using a standardized extraction form developed for this review. Extracted information included: author(s), year of publication, country or region, study design, sample characteristics, definitions and measures of supportive care, maternal satisfaction outcomes and key findings. Data extraction was conducted independently by two reviewers to ensure accuracy and completeness. Authors were not contacted for additional information.
Data items	10a	List and define all outcomes for which data were sought. Specify whether all results that were compatible with each outcome domain in each study were sought (e.g. for all measures, time points, analyses), and if not, the methods used to decide which results to collect.	Main outcome: maternal childbirth satisfaction- Women's subjective evaluation of childbirth experiences including emotional, psychological, interpersonal and physical dimensions. Acceptable measurement instruments such as validated scales, structured questionnaires, interviews and qualitative narratives. Time point as it was reported in each study. For example, intrapartum, postpartum etc. Additional outcomes: types/components of supportive care- forms of emotional, physical, advocacy etc. Instruments; observational interviews, labor support scales, surveys etc. Effect measure- narrative synthesis and descriptive; associations between supportive care and maternal satisfaction- which elements of support, eg informational re4assurance, communication quality etc are linked to higher satisfaction. Measurement instruments- quantitative correlations, regressions, thematic findings etc. Effect- Descriptive summary of reported associations; mechanisms linking supportive care to satisfaction-.psychological, emotional etc associations through which supportive care enhances satisfaction. For example; reduced fear, anxiety etc. Measurement; qualitative, thematic, mixed integrations etc. Effect; Narrative synthesis. Negative or unsupported care experiences leading to dissatisfaction- lack of support, disrespect, mistreatment, neglect etc impact on childbirth satisfaction. Measurements- qualitative narratives, observational studies etc. Effects; Narrative synthesis
	10b	List and define all other variables for which data were sought (e.g. participant and intervention characteristics, funding sources). Describe any assumptions made about any missing or	Participant characteristics — maternal age, parity, sociodemographic details, and relevant clinical context.<br><br>Study setting — country, type of maternity unit, and model of care.<br><br>Intervention/support characteristics — type of supportive care provided (emotional, informational, physical, or continuous support).<br><br>Study design characteristics — qualitative, quantitative, or mixed-methods features relevant to interpretation.

Section and Topic	Item #	Checklist item	Location where item is reported
		unclear information.	Funding sources — when reported, to identify potential conflicts of interest.<br><br>When information was missing or unclear, no assumptions were made. Variables not explicitly reported in a study were recorded as "not reported." Ambiguous descriptions were clarified only when supported by contextual information within the study; otherwise, they were left as unclear without imputation or inference.
Study risk of bias assessment	11	Specify the methods used to assess risk of bias in the included studies, including details of the tool(s) used, how many reviewers assessed each study and whether they worked independently, and if applicable, details of automation tools used in the process.	Risk of bias was assessed using the Mixed Methods Appraisal Tool (MMAT), which is designed to appraise qualitative, quantitative, and mixed-methods studies within a single framework. Assessment was conducted by one reviewer by applying the MMAT criteria to each included study, assessing methodological quality across the relevant domains for the study design and was checked by another reviewer. Any disagreements were resolved through discussion, with a third reviewer consulted when necessary. No automation tools were used during the risk-of-bias assessment process.
Effect measures	12	Specify for each outcome the effect measure(s) (e.g. risk ratio, mean difference) used in the synthesis or presentation of results.	Narrative synthesis planned for qualitative and mixed-methods studies. Quantitative findings will be summarised descriptively using reported associations, proportions, or mean scores. For all outcomes, results were synthesised descriptively. The primary effect measures extracted and reported were:<br><br>Maternal satisfaction scores — presented as mean scores, proportions, or narrative descriptions depending on how each study reported the outcome.<br>Childbirth experience measures — extracted as mean scale scores, thematic findings, or qualitative summaries.<br>Supportive care indicators — reported as frequencies, descriptive statistics, or qualitative accounts.<br>Because the review did not conduct a meta-analysis, no pooled effect measures (e.g., risk ratios, mean differences) were calculated. All results compatible with each outcome domain were included regardless of measurement tool or scale format.
Synthesis methods	13a	Describe the processes used to decide which studies were eligible for each synthesis (e.g. tabulating the study intervention characteristics and comparing against the planned groups for each synthesis (item #5)).	Studies will be grouped according to supportive care components, maternal satisfaction outcomes, childbirth outcomes or experiences and study design. To determine which studies were eligible for each synthesis, the characteristics of all included studies were first tabulated, including population, setting, type of supportive care, and reported outcomes. These characteristics were compared against the predefined outcome domains and conceptual groupings specified in the review protocol. Studies were then allocated to each synthesis based on whether they reported data relevant to the outcome of interest—specifically maternal satisfaction, childbirth experience, or supportive care received. All studies reporting at least one relevant outcome were included in the corresponding synthesis. No automation tools were used in this process.
	13b	Describe any methods required to prepare	Methods used to prepare data for synthesis included checking extracted values for consistency and completeness across studies. When studies reported outcomes using different formats or scales within the same outcome domain, results were standardised

PRISMA

PRISMA 2020 Checklist

Section and Topic	Item #	Checklist item	Location where item is reported
		the data for presentation or synthesis, such as handling of missing summary statistics, or data conversions.	conceptually to ensure comparability across studies. No statistical conversions, imputations, or transformations were performed, and no attempts were made to estimate missing summary statistics. When multiple time points or measures were reported, the reviewers selected the result most closely aligned with the predefined outcome definition. No automation tools were used in data preparation.
	13c	Describe any methods used to tabulate or visually display results of individual studies and syntheses.	Results from individual studies were tabulated in structured summary tables, which included study characteristics, participant details, types of supportive care, and all relevant outcomes. To support interpretation, several tables were grouped according to the three research questions, allowing findings to be organised and compared within each conceptual focus of the review.<br>To complement the tabulated data, VOSviewer (VOS) was used to generate visual thematic cluster maps, illustrating conceptual relationships and co-occurring themes across the included studies. These visualisations supported the narrative synthesis by highlighting dominant thematic patterns and how they clustered within the literature.<br>Because no meta-analysis was conducted, no statistical plots (e.g., forest plots) were produced. Presentation of results relied on structured tables and VOS-based visual maps to summarise and communicate the findings clearly.
	13d	Describe any methods used to synthesize results and provide a rationale for the choice(s). If meta-analysis was performed, describe the model(s), method(s) to identify the presence and extent of statistical heterogeneity, and software package(s) used.	Results were synthesised using a narrative and thematic synthesis approach, appropriate for the diverse study designs and heterogeneous outcome measures included in the review. Because the studies varied substantially in methodology, measurement tools, and reporting formats, meta-analysis was not feasible, and no statistical pooling was attempted.<br><br>The synthesis process involved:<br>Grouping studies according to the three predefined research questions to ensure alignment between evidence and review objectives.<br>Tabulating key findings from individual studies to enable structured comparison across outcome domains.<br>Thematic synthesis to identify recurring concepts related to maternal satisfaction, childbirth experience, and supportive care.<br>Use of VOSviewer (VOS) to generate visual thematic cluster maps, which supported the identification of conceptual relationships and strengthened the interpretive synthesis.<br>Because no meta-analysis was conducted:<br>No statistical models were applied.<br>No measures of heterogeneity (e.g., I^2, τ^2) were calculated.<br>No statistical software for meta-analysis was used.<br>The chosen synthesis methods were justified by the qualitative nature of much of the included evidence, the variability in outcome measurement, and the aim to explore thematic patterns rather than estimate pooled effect sizes.
	13e	Describe any methods used to explore possible causes of heterogeneity among study results (e.g. subgroup analysis, meta-regression).	No formal statistical methods were used to explore heterogeneity (such as subgroup analysis or meta-regression), as a meta-analysis was not conducted. The included studies varied substantially in design, measurement tools, outcome definitions, and reporting formats, making quantitative pooling inappropriate.<br>Instead, potential sources of heterogeneity were explored qualitatively through:<br>Narrative comparison of study characteristics, including setting, population, and type of supportive care.<br>Thematic grouping of findings according to the three research questions.<br>VOSviewer-generated thematic cluster maps, which visually highlighted conceptual differences and similarities across studies.<br>These approaches allowed heterogeneity to be examined descriptively, consistent with the aims and methodological diversity of the included evidence.

PRISMA

PRISMA 2020 Checklist

Section and Topic	Item #	Checklist item	Location where item is reported
	13f	Describe any sensitivity analyses conducted to assess robustness of the synthesized results.	No formal statistical sensitivity analyses were conducted because a meta-analysis was not performed. The included studies differed substantially in design, outcome measures, and reporting formats, making quantitative sensitivity testing inappropriate.<br>Instead, the robustness of the synthesized results was explored qualitatively through:<br>Comparing findings across study designs to assess whether conclusions were consistent between qualitative, quantitative, and mixed-methods evidence.<br>Examining the influence of lower-quality studies (as assessed by MMAT) by checking whether excluding them would alter the thematic patterns identified.<br>Cross-checking themes against VOSviewer-generated clusters to ensure that key themes were supported by multiple studies rather than driven by single outliers.<br>These qualitative checks indicated that the main themes and interpretations were stable and did not depend on any single study or subgroup of studies.
Reporting bias assessment	14	Describe any methods used to assess risk of bias due to missing results in a synthesis (arising from reporting biases).	No formal statistical methods were used to assess risk of bias due to missing results, as no meta-analysis was conducted and therefore techniques such as funnel plots or regression-based tests for small-study effects were not applicable.<br>Instead, potential reporting biases were considered qualitatively by:<br>Comparing reported outcomes across studies to identify whether expected outcomes (e.g., maternal satisfaction, childbirth experience, supportive care) were selectively omitted.<br>Reviewing study protocols or methods sections when available, to check for discrepancies between planned and reported outcomes.<br>Assessing completeness of outcome reporting within each study, noting instances where outcomes were mentioned but not fully presented.<br>Considering the influence of missing or selectively reported results during the narrative synthesis, ensuring that interpretations did not rely on single studies with incomplete reporting.<br>Because the evidence base included diverse qualitative, quantitative, and mixed-methods studies, these descriptive checks were the most appropriate way to evaluate the potential for reporting bias.
Certainty assessment	15	Describe any methods used to assess certainty (or confidence) in the body of evidence for an outcome.	No formal certainty-of-evidence framework (e.g., GRADE or CERQual) was applied in this review due to heterogeneity in study designs and the use of narrative synthesis. Findings were interpreted based on the methodological quality of included studies assessed using MMAT.
**RESULTS**			
Study selection	16a	Describe the results of the search and selection process, from the number of records identified in the search to the number of studies included in the review, ideally using a flow diagram.	The search identified a total of 546 records. After removal of duplicates, 391 records were screened by title and abstract, of which 209 were excluded. 182 full-text articles were assessed for eligibility, and 157 were excluded with reasons. A total of 25 studies met the inclusion criteria and were included in the final review.

Section and Topic	Item #	Checklist item	Location where item is reported
	16b	Cite studies that might appear to meet the inclusion criteria, but which were excluded, and explain why they were excluded.	Several studies initially appeared to meet the inclusion criteria based on title and abstract screening but were excluded after full-text assessment. Reasons for exclusion are summarised below:<br>Power et al. 2023 (Qualitative interview study) — Does a mother's childbirth experience influence her perceptions of her baby's behaviour?<br>Excluded because the study focused on maternal perceptions of infant behaviour, not on maternal satisfaction, childbirth experience, or supportive care during labour.<br>Friedman et al.2020 (Social support & antepartum depression) — Association of social support and antepartum depression among pregnant women<br>Excluded because it examined antepartum depression and general social support during pregnancy, not intrapartum supportive care or childbirth experience.<br>Topçu et al. 2025 (Postpartum control & support) — Investigating Maternal Perception of Control and Support in the Postpartum Period<br>Excluded because the study assessed postpartum perceptions, whereas the review focused on supportive care and experiences during labour and birth.<br>Lochmannová & Martin 2025 (Psychometric study) — Exploring the Role of Social Support in Postpartum Well-Being<br>Excluded because it was a psychometric validation study and examined postpartum well-being, not intrapartum supportive care or childbirth experience.<br>These studies were therefore excluded because they did not address the population, timing, or outcome domains specified in the review's eligibility criteria.
Study characteristics	17	Cite each included study and present its characteristics.	The review included 25 studies, representing a wide range of geographical regions, methodological approaches, and supportive-care models. Full details of each study are presented in Tables 3, and a narrative overview is provided below.<br><br>Study Designs and Settings<br>The included studies comprised:<br><br>Randomized controlled trials (RCTs) (n = 4)<br><br>Quasi-experimental or non-randomised studies (n = 3)<br><br>Cross-sectional surveys (n = 11)<br><br>Qualitative studies (n = 6)<br><br>Mixed-methods studies (n = 2)<br><br>Studies were conducted across Europe, Asia, Africa, and the Middle East, with particularly strong representation from Turkey, Sweden, China, Kenya, and the Netherlands. Settings included hospital labour wards, maternity units, and community-based doula

Section and Topic	Item #	Checklist item	Location where item is reported
			programmes.<br><br>Participants<br>Sample sizes ranged from 10 qualitative participants to over 12,000 survey respondents. Most studies involved:<br><br>Labouring or postpartum women<br><br>Primiparous or primigravida women<br><br>Migrant women<br><br>Support persons (in a small number of studies)<br><br>Types of Support Examined<br>Across the 25 studies, supportive care fell into several recurring categories:<br><br>Continuous emotional and physical support (e.g., doula care, one-to-one midwifery support)<br><br>Companion support (partners, family members, lay companions)<br><br>Informational and communication-based support (e.g., empathic communication training, respectful maternity care frameworks)<br><br>Advocacy and cultural mediation (especially in migrant-focused doula programmes)<br><br>Environmental or structural support (e.g., single vs shared labour rooms, involvement of support persons in decision-making)<br><br>Outcomes Reported<br>Most studies reported outcomes related to:<br><br>Maternal satisfaction<br><br>Childbirth experience<br><br>Fear, anxiety, or emotional well-being

Section and Topic	Item #	Checklist item	Location where item is reported
			Perceived autonomy, dignity, and respectful care<br>Labour progress and clinical outcomes (in a smaller subset of studies)<br>Across designs, supportive care was consistently associated with:<br>Reduced fear and anxiety<br>Improved coping and emotional security<br>Enhanced satisfaction with childbirth<br>Better communication and shared decision-making<br>Increased trust in providers<br>Improved perceptions of dignity, respect, and autonomy<br>In some RCTs, shorter labour duration and fewer interventions<br>Overall Characteristics<br>The evidence base reflects substantial methodological diversity, with studies ranging from small qualitative explorations of women's experiences to large national surveys assessing respectful maternity care. Despite this variation, the studies collectively highlight the importance of continuous, compassionate, and communicative support in shaping positive childbirth experiences.
Risk of bias in studies	18	Present assessments of risk of bias for each included study.	The methodological quality of the 25 included studies was assessed using the Mixed Methods Appraisal Tool (MMAT) based on study design *(Table 3: Appendix supplementary file)*. The MMAT was selected because it allows appraisal of qualitative, quantitative, and mixed-methods studies within a single framework. Given the heterogeneity of included qualitative, quantitative, and mixed-methods studies, MMAT was selected to ensure consistent appraisal across study designs. MMAT was used to assess methodological quality at the study level. This does not represent a formal assessment of certainty of evidence, and no GRADE or CERQual framework was applied.<br>Quality appraisal was conducted by one reviewer and checked independently by a second reviewer, with any discrepancies resolved through discussion to ensure consistency and accuracy. Overall, the included studies demonstrated generally good methodological quality across qualitative, quantitative, and mixed-methods designs. Most studies met the majority of MMAT criteria, indicating acceptable methodological rigor. Common methodological strengths included appropriate study designs, adequate sampling strategies, and use of validated measurement tools. Some studies exhibited limitations, particularly related to randomisation procedures, blinding, and control of confounding in quantitative designs, as well as limited reflexivity reporting in a small number of qualitative studies.

Section and Topic	Item #	Checklist item	Location where item is reported
			No studies were excluded based on methodological quality, as all met the minimum eligibility criteria for inclusion. The results of the MMAT appraisal were used to inform interpretation of findings rather than to generate exclusion or strict categorical quality rankings. During narrative synthesis, findings were interpreted in light of study design, methodological quality, and consistency across studies. Greater emphasis was placed on recurring findings observed across multiple studies and study designs rather than on individual studies alone. Where studies had methodological limitations, these were considered during synthesis and discussion of the evidence base.<br>Because several studies addressed multiple dimensions of supportive care, findings were sometimes synthesised across overlapping themes depending on their relevance to specific research questions. This approach ensured that all relevant evidence was incorporated while maintaining alignment with the review objectives
Results of individual studies	19	For all outcomes, present, for each study: (a) summary statistics for each group (where appropriate) and (b) an effect estimate and its precision (e.g. confidence/credible interval), ideally using structured tables or plots.	For each included study, summary statistics for relevant outcomes (e.g. measures of maternal satisfaction, childbirth experience, fear/anxiety, respectful care, and labour outcomes) were extracted and presented in structured summary tables (Tables 3). Where reported, we recorded group-specific values (e.g. means and standard deviations, proportions, or medians and interquartile ranges) for intervention versus comparison groups (such as continuous support vs standard care, doula vs routine care, or presence vs absence of a companion).<br>Because the review primarily used a narrative synthesis and the included studies were heterogeneous in design, measures, and reporting formats, formal effect estimates with confidence intervals were not consistently available or directly comparable across studies.
Results of syntheses	20a	For each synthesis, briefly summarise the characteristics and risk of bias among contributing studies.	The methodological quality of the 25 included studies was assessed using the Mixed Methods Appraisal Tool (MMAT) based on study design *(Table 3: Appendix supplementary file)*. The MMAT was selected because it allows appraisal of qualitative, quantitative, and mixed-methods studies within a single framework. Given the heterogeneity of included qualitative, quantitative, and mixed-methods studies, MMAT was selected to ensure consistent appraisal across study designs. MMAT was used to assess methodological quality at the study level. This does not represent a formal assessment of certainty of evidence, and no GRADE or CERQual framework was applied.<br>Quality appraisal was conducted by one reviewer and checked independently by a second reviewer, with any discrepancies resolved through discussion to ensure consistency and accuracy. Overall, the included studies demonstrated generally good methodological quality across qualitative, quantitative, and mixed-methods designs. Most studies met the majority of MMAT criteria, indicating acceptable methodological rigor. Common methodological strengths included appropriate study designs, adequate sampling strategies, and use of validated measurement tools. Some studies exhibited limitations, particularly related to randomisation procedures, blinding, and control of confounding in quantitative designs, as well as limited reflexivity reporting in a small number of qualitative studies.<br>No studies were excluded based on methodological quality, as all met the minimum eligibility criteria for inclusion. The results of the MMAT appraisal were used to inform interpretation of findings rather than to generate exclusion or strict categorical quality rankings. During narrative synthesis, findings were interpreted in light of study design, methodological quality, and consistency across studies. Greater emphasis was placed on recurring findings observed across multiple studies and study designs rather than on individual studies alone. Where studies had methodological limitations, these were considered during synthesis and discussion of the evidence base.<br>Because several studies addressed multiple dimensions of supportive care, findings were sometimes synthesised across overlapping themes depending on their relevance to specific research questions. This approach ensured that all relevant evidence was incorporated while maintaining alignment with the review objectives.

Section and Topic	Item #	Checklist item	Location where item is reported
	20b	Present results of all statistical syntheses conducted. If meta-analysis was done, present for each the summary estimate and its precision (e.g. confidence/credible interval) and measures of statistical heterogeneity. If comparing groups, describe the direction of the effect.	No formal statistical syntheses or meta-analyses were conducted in this review. The included studies were highly heterogeneous in terms of design (RCTs, quasi-experimental, cross-sectional, qualitative, mixed-methods), outcome measures, instruments, and reporting formats, which made quantitative pooling inappropriate.<br>Instead, results were synthesised narratively for each outcome domain (emotional outcomes, childbirth experience/satisfaction, and respectful/person-centred maternity care). For randomized and quasi-experimental studies, we described the direction of effects (e.g. continuous support vs standard care, doula vs routine care, presence vs absence of a companion), consistently showing that supportive care was associated with:<br>Reduced fear and anxiety<br>Improved coping and emotional well-being<br>Higher satisfaction and more positive birth experiences<br>Better autonomy, communication, and respectful care<br>Because no meta-analysis was performed, no pooled summary estimates, confidence intervals, or measures of statistical heterogeneity (e.g. I^2, τ^2) are reported.
	20c	Present results of all investigations of possible causes of heterogeneity among study results.	No formal statistical investigations of heterogeneity were conducted because a meta-analysis was not performed. The included studies differed substantially in design, populations, outcome measures, and types of supportive care, making quantitative exploration of heterogeneity inappropriate.<br>Instead, potential sources of heterogeneity were examined qualitatively during the narrative synthesis. Differences in findings were explored in relation to:<br>Study design (e.g., RCTs vs cross-sectional vs qualitative studies)<br>Type of supportive care (continuous support, companion support, doula care, communication-based interventions)<br>Population characteristics (primiparous women, migrant women, postpartum women)<br>Setting and health-system context (high-income vs low-resource settings, hospital vs community-based care)<br>Measurement tools and outcome definitions (different scales for satisfaction, fear, respectful care)<br>Across these dimensions, variations in study context and methodology helped explain differences in reported outcomes. For example, studies involving continuous one-to-one support tended to show stronger improvements in emotional outcomes than studies examining structural or environmental factors (e.g., shared vs single labour rooms). Similarly, studies conducted in low-resource settings often highlighted issues of mistreatment and communication gaps, which shaped the magnitude and direction of effects.<br>These qualitative comparisons provided insight into the likely sources of heterogeneity, even though no statistical tests were performed.
	20d	Present results of all sensitivity analyses conducted to assess the robustness of the synthesized results.	No formal sensitivity analyses were conducted because no meta-analysis or statistical synthesis was performed. The included studies were highly heterogeneous in design, populations, outcome measures, and reporting formats, making quantitative sensitivity testing inappropriate.<br>Instead, the robustness of the narrative findings was explored qualitatively by:<br><br>Considering whether conclusions were dependent on specific study designs (e.g., RCTs vs cross-sectional studies)<br>Examining whether findings were consistent across different settings and populations<br>Assessing whether studies with higher risk of bias produced results that differed meaningfully from those with lower risk<br>Reviewing whether the direction of findings remained stable across various types of supportive care (continuous support, doula care,

Section and Topic	Item #	Checklist item	Location where item is reported
			companion support, communication-based interventions)<br>These qualitative checks indicated that the overall conclusions were not driven by any single study or study type, and the direction of effects remained consistent across the evidence base.
Reporting biases	21	Present assessments of risk of bias due to missing results (arising from reporting biases) for each synthesis assessed.	No formal statistical assessment of reporting bias (such as funnel plots or small-study effect tests) was conducted because no meta-analysis was performed and the included studies used heterogeneous designs and outcome measures.<br>Across the narrative syntheses, we assessed the possibility of reporting bias qualitatively. The included studies did not provide sufficient information to determine whether any outcomes were selectively withheld, and most studies reported the outcomes relevant to their stated aims. Because of the diversity of study designs (RCTs, quasi-experimental, cross-sectional, qualitative, and mixed-methods), it was not possible to systematically evaluate reporting bias in a uniform way.<br>Overall, there was no clear evidence of reporting bias, but the possibility of missing or selectively reported results cannot be entirely excluded, as is typical in reviews of heterogeneous primary studies.
Certainty of evidence	22	Present assessments of certainty (or confidence) in the body of evidence for each outcome assessed.	The review used a narrative synthesis and the included studies were methodologically diverse, a formal GRADE assessment with quantitative ratings (e.g., “high,” “moderate,” “low,” “very low”) was not appropriate. Instead, the certainty of the evidence for each outcome was assessed qualitatively, taking into account study design, consistency of findings, and relevance to the review questions.<br>Emotional Outcomes (fear, anxiety, coping, emotional well-being)<br>The certainty of evidence for emotional outcomes was judged as moderate. Although study designs varied, findings were highly consistent: continuous support, doula care, and companion involvement were repeatedly associated with reduced fear and anxiety and improved coping. RCTs contributed stronger evidence, while cross-sectional and qualitative studies supported the direction of effect.<br>Childbirth Experience and Maternal Satisfaction<br>The certainty of evidence for childbirth experience and satisfaction was assessed as moderate. Multiple study types (RCTs, cross-sectional surveys, qualitative studies, mixed-methods) consistently showed that supportive care improved satisfaction, perceived control, and overall experience. Variation in measurement tools reduced comparability, but the direction of findings was stable across contexts.<br>Respectful and Person-Centred Maternity Care<br>The certainty of evidence for respectful care outcomes was considered moderate to low. Evidence came largely from cross-sectional and qualitative studies, which provided rich descriptions but lacked experimental control. However, findings were consistent: supportive care, especially continuous presence, communication, and advocacy—was associated with improved autonomy, dignity, and respectful treatment.<br>Clinical Outcomes (labour duration, interventions, breastfeeding initiation)<br>The certainty of evidence for clinical outcomes was low, as only a small number of RCTs and quasi-experimental studies reported these outcomes. Results generally favoured supportive care (e.g., shorter labour, fewer interventions), but the evidence base was limited in size and heterogeneity.<br>Overall Certainty Across Outcomes<br>Across all outcome domains, the evidence was strengthened by:<br><br>Consistency of findings across diverse settings and study designs<br>Converging evidence from both quantitative and qualitative studies<br>Relevance to real-world maternity care contexts<br>Certainty was limited by:

Section and Topic	Item #	Checklist item	Location where item is reported
			Variation in outcome measures<br>Non-experimental designs in many studies<br>Differences in reporting detail<br>Overall, the body of evidence provides moderate confidence that supportive care during labour improves emotional well-being, childbirth experience, and respectful maternity care.
**DISCUSSION**			
Discussion	23a	Provide a general interpretation of the results in the context of other evidence.	The findings of this review align closely with the broader body of international evidence demonstrating that supportive care during labour, whether provided by midwives, doulas, partners, or trained companions, has a consistently positive influence on women's emotional well-being, childbirth experience, and perceptions of respectful maternity care. Across diverse settings and study designs, the included studies showed that continuous presence, emotional reassurance, effective communication, and advocacy contribute to reduced fear and anxiety, improved coping, and higher satisfaction with childbirth. These results reinforce long-standing conclusions from global research, including the WHO recommendations on intrapartum care, which emphasise continuous support as a core component of high-quality, person-centred maternity care.<br>The review also supports evidence from previous observational and qualitative studies showing that supportive care enhances women's sense of autonomy, dignity, and trust in providers. This is particularly consistent with research from low-resource settings, where communication gaps and mistreatment are more frequently reported. The present findings extend this evidence by demonstrating that supportive care can mitigate negative experiences even in constrained environments, highlighting its relevance across health-system contexts.<br>Although only a small number of included studies examined clinical outcomes, the direction of effects such as shorter labour duration, fewer interventions, and improved breastfeeding initiation, mirrors earlier trials and Cochrane reviews showing that continuous support can improve both emotional and physiological outcomes. The consistency of these findings across different populations, including migrant women and primiparous women, suggests that supportive care is broadly beneficial and adaptable to varying cultural and organisational contexts.<br>Taken together, the results of this review contribute to a growing international consensus that supportive care is a critical determinant of positive childbirth experiences. They underscore the importance of integrating emotional, informational, and physical support into routine maternity care and highlight the need for health systems to prioritise models that enable continuous, compassionate, and person-centred support during labour.
	23b	Discuss any limitations of the evidence included in the review.	This review has several limitations. First, restricting the search to English-language publications may have excluded relevant studies from non-English-speaking contexts, which could limit the global representativeness of the findings. Second, variation in how supportive care was defined and measured across studies made direct comparison difficult. The use of narrative synthesis, although appropriate for heterogeneous evidence, did not allow for statistical pooling or the calculation of effect sizes. As a result, the strength of associations could not be quantified. Heterogeneity in study designs, measurement tools, and definitions of supportive care also limited comparability across studies. In addition, most included studies were conducted in facility-based settings, which may reduce the transferability of findings to community or home-birth contexts. Finally, the predominance of cross-sectional designs limits conclusions about the long-term effects of supportive care on women's wellbeing and trust in the health system.<br>Future research should examine how supportive care is implemented and sustained within constrained health systems. It should also explore its mechanisms across different sociocultural contexts. Studies using standardized measures and longitudinal designs are needed to better understand the longer-term associations of supportive care with women's experiences and outcomes.
	23c	Discuss any limitations of the	Several limitations of the review processes should be acknowledged. First, although a comprehensive search strategy was used, the review was limited to studies published in English, which may have excluded relevant evidence from non-English-speaking contexts.

PRISMA 2020 Checklist

Section and Topic	Item #	Checklist item	Location where item is reported
		review processes used.	Second, because the included studies used diverse designs and outcome measures, it was not possible to conduct a meta-analysis; therefore, the synthesis relied on narrative methods, which may introduce subjectivity in interpretation. Third, data extraction and synthesis were constrained by the level of detail reported in the primary studies, particularly for observational and qualitative designs, which varied in methodological transparency.<br>Additionally, the heterogeneity of study designs (RCTs, cross-sectional surveys, qualitative studies, mixed-methods) meant that a uniform approach to assessing risk of bias and certainty of evidence was not feasible. While efforts were made to apply consistent criteria, differences in reporting standards across study types may have influenced the depth of appraisal. Finally, the review did not include unpublished studies or grey literature, which may increase the risk of publication bias, although the extent of this bias could not be formally assessed.<br>Overall, these limitations reflect common challenges in synthesising complex, mixed-method evidence and should be considered when interpreting the findings.
	23d	Discuss implications of the results for practice, policy, and future research.	The findings of this review highlight several important implications for maternity care practice. First, the consistent association between supportive care and women's evaluations of childbirth highlights the importance of emotional reassurance, respectful communication, and continuous presence within routine intrapartum care. These practices do not require advanced technology or additional clinical resources, making them feasible across a wide range of facility-based settings. Second, the review underscores the value of enabling women to have a chosen companion during labour. Studies reporting on companionship indicated that women often felt more secure, informed, and emotionally supported when accompanied by a trusted individual. Policies that facilitate companion presence can support women's sense of safety and involvement in their care. Third, communication emerged as a central determinant of women's perceptions of maternity care. Interventions that strengthen empathic communication such as provider training programmes were associated with improvements in women's reported experiences. Integrating communication training into professional development for midwives, nurses, and obstetric staff may support interpersonal care and improve women's reported experiences. Finally, the review revealed that postpartum support should be prioritised alongside intrapartum care. Women's reflections on childbirth were closely linked to whether they felt listened to, respected, and supported after birth. Structured debriefing, follow-up communication, and accessible informational tools can support women's reflection on their experiences and emotional wellbeing.
**OTHER INFORMATION**			
Registration and protocol	24a	Provide registration information for the review, including register name and registration number, or state that the review was not registered.	Registered in PROSPERO: CRD420261385103
	24b	Indicate where the review protocol can be accessed, or state that a protocol was not prepared.	A full protocol has been written but is not available because the protocol is a working document developed for internal use and has not undergone formatting or any peer review for public release.
	24c	Describe and explain any amendments to information provided at registration or in the protocol.	No amendment reported

PRISMA

PRISMA 2020 Checklist

Section and Topic	Item #	Checklist item	Location where item is reported
Support	25	Describe sources of financial or non-financial support for the review, and the role of the funders or sponsors in the review.	This work was supported by the National Natural Science Foundation of China (Grant No.71974079) awarded to (ZL). The funding body had no role in the study design, data collection, analysis, interpretation of findings, manuscript preparation, or decision to publish.
Competing interests	26	Declare any competing interests of review authors.	There are no competing interests of review authors
Availability of data, code and other materials	27	Report which of the following are publicly available and where they can be found: template data collection forms; data extracted from included studies; data used for all analyses; analytic code; any other materials used in the review.	Template data collection forms — The standardized data extraction form used in this review is not publicly available but can be provided upon reasonable request from the corresponding author.<br>Data extracted from included studies — The extracted dataset summarizing study characteristics and outcomes is included in the manuscript tables and supplementary material. No additional unpublished extracted data are available.<br>Data used for all analyses — All data used in the analyses are fully presented within the Results section and supplementary tables. No separate dataset is publicly archived.<br>Analytic code — This review did not use custom analytic code; therefore, no analytic code is available. All bibliometric visualisations and co-occurrence analyses were generated using VOSviewer internal algorithms and standard processing functions.<br>Other materials used in the review — No additional materials were produced or used beyond those reported in the manuscript

From: Page MJ, McKenzie JE, Bossuyt PM, Boutron I, Hoffmann TC, Mulrow CD, et al. The PRISMA 2020 statement: an updated guideline for reporting systematic reviews. BMJ 2021;372:n71. doi: 10.1136/bmj.n71.
This work is licensed under CC BY 4.0. To view a copy of this license, visit https://creativecommons.org/licenses/by/4.0/
